# Different Hydrophobins of *Fusarium graminearum* Are Involved in Hyphal Growth, Attachment, Water-Air Interface Penetration and Plant Infection

**DOI:** 10.3389/fmicb.2019.00751

**Published:** 2019-04-12

**Authors:** Alessandra Quarantin, Birgit Hadeler, Cathrin Kröger, Wilhelm Schäfer, Francesco Favaron, Luca Sella, Ana Lilia Martínez-Rocha

**Affiliations:** ^1^Molekulare Phytopathologie, Institut für Pflanzenwissenschaften und Mikrobiologie, Universität Hamburg, Hamburg, Germany; ^2^Dipartimento Territorio e Sistemi Agro-Forestali (TESAF), Università degli Studi di Padova, Padova, Italy

**Keywords:** *Fusarium graminearum*, hydrophobin, attachment, water-air interface, virulence

## Abstract

Hydrophobins (HPs) are small secreted fungal proteins possibly involved in several processes such as formation of fungal aerial structures, attachment to hydrophobic surfaces, interaction with the environment and protection against the host defense system. The genome of the necrotrophic plant pathogen *Fusarium graminearum* contains five genes encoding for HPs (FgHyd1-5). Single and triple FgHyd mutants were produced and characterized. A reduced growth was observed when the Δ*Fghyd2* and the three triple mutants including the deletion of FgHyd2 were grown in complete or minimal medium. Surprisingly, the growth of these mutants was similar to wild-type when grown under ionic, osmotic or oxidative stress conditions. All the mutant strains confirmed the ability to develop conidia and perithecia, suggesting that the FgHyds are not involved in normal development of asexual and sexual structures. A reduction in the ability of hyphae to penetrate through the water-air interface was observed for the single mutants Δ*Fghyd2* and Δ*Fghyd3* as well as for the triple mutants including the deletion of FgHyd2 and FgHyd3. Besides, Δ*Fghyd3* and the triple mutant Δ*Fghyd234* were also affected in the attachment to hydrophobic surface. Indeed, wheat infection experiments showed a reduction of symptomatic spikelets for Δ*Fghyd2* and Δ*Fghyd3* and the triple mutants only when spray inoculation was performed. This result could be ascribed to the affected ability of mutants deleted of FgHyd2 and FgHyd3 to penetrate through the water-air interface and to attach to hydrophobic surfaces such as the spike tissue. This hypothesis is strengthened by a histological analysis, performed by fluorescence microscopy, showing no defects in the morphology of infection structures produced by mutant strains. Interestingly, triple hydrophobin mutants were significantly more inhibited than wild-type by the treatment with a systemic triazole fungicide, while no defects at the cell wall level were observed.

## Introduction

Hydrophobins (HPs) are small secreted cysteine-rich amphiphilic proteins, found only in filamentous fungi (Wessels, 2000; Wösten, 2001). The name hydrophobin was coined after the examination of genes encoding small hydrophobic proteins expressed during fruiting body formation in *Schizophyllum commune* (Wessels et al., 1991a,b). After that, hydrophobins have been identified in several other fungi (Lauter et al., 1992; Beckerman and Ebbole, 1996; Segers et al., 1999; Fuchs et al., 2004; Izumitsu et al., 2010; Heddergott et al., 2012; Valsecchi et al., 2018).

Hydrophobins are characterized by the presence of eight cysteine residues in their amino acid sequences which are arranged in a conserved pattern and form four disulphide bridges. Nevertheless, HP amino acid sequences usually show a low structural similarity level. HPs have been isolated from Ascomycetes, Basidiomycetes, and Zygomycetes as well, and, according to hydropathy patterns and solubility characteristics, are divided into two classes. Class I HPs have been identified in both Ascomycetes and Basidiomycetes while Class II HPs have been found only in Ascomycetes (Wessels, 1994). The two classes can also be distinguished by the amino acids spacing between the cysteine residues, which is more conserved in the Class II HPs (Kershaw and Talbot, 1998; Whiteford and Spanu, 2002). Additionally, in *Aspergillus* species an intermediate Class III has been described (Jensen et al., 2010; Littlejohn et al., 2012).

Secreted as protein monomers, HPs are able to self-assemble at water-air interfaces in response to the environment and to aggregate to amphipathic membranes (Wösten et al., 1999; Wösten and Scholtmeijer, 2015). Although proteins of either Classes are able to form stable aggregates, those of the Class I can only be dissolved by strong acids (Wessels et al., 1991a; de Vries et al., 1993), while those of the Class II can be easily dissolved in aqueous dilutions of organic solvents (Wösten and de Vocht, 2000). The event triggering the formation of highly stable aggregates of the Class I hydrophobins, which are similar to amyloid fibrils, is the destabilization of the specific disulfide bond loop L1 (Pennacchio et al., 2018). In contrast, monolayer aggregates of the Class II hydrophobins require the disulfide bonds for protein structural stability (Sallada et al., 2018).

By self-assembling at the water-air interfaces (Wösten and de Vocht, 2000), HPs would allow fungi to escape the aqueous environment. Indeed, HPs coat the surface of the hydrophilic cell wall polysaccharides of conidia, spores, hyphae and fruiting structures (Wessels, 1997; Wösten et al., 1999, Wösten, 2001), and expose their hydrophobic layer to the outside, conferring water-repellent properties to these fungal surfaces (Wösten et al., 1994; Kershaw and Talbot, 1998; Wösten, 2001). In fact, several null-hydrophobin fungal mutants show “easily wettable” phenotypes, indicating that HPs confer surface hydrophobicity to aerial hyphae and spores (Talbot et al., 1993; Mosbach et al., 2011; Heddergott et al., 2012).

In conidia and hyphae, this hydrophobic coating has been also proposed to have a protecting role both against desiccation and wetting, also aiding dispersal of conidia (Wösten, 2001; Whiteford and Spanu, 2001, 2002; Klimes and Dobinson, 2006). In addition, surface rodlet layer of airborne conidia prevents immunorecognition by both innate and adaptative immune defense systems (Aimanianda et al., 2009; Heddergott et al., 2012; Voltersen et al., 2018). Furthermore, HPs can also be involved in many morphogenetic processes, including conidia germination, fruit body development, infection structure formation, attachment of fungi to solid supports and fungal pathogenicity (Talbot et al., 1996; Whiteford and Spanu, 2001; Kim et al., 2005; Klimes and Dobinson, 2006).

The role of HPs has been characterized in several fungal pathogens. The Class I Mpg1 hydrophobin of the rice blast fungus *Magnaporthe grisea* is important for efficient conidiogenesis and for pathogenicity on host plants (Talbot et al., 1993) and is required for attachment to the leaf surface (Talbot et al., 1996; Whiteford and Spanu, 2002). Indeed, the Δmpg1 mutant has impaired ability to form appressoria, probably due to the inability of the germ tubes to firmly attach to the hydrophobic plant cuticle and to appropriately sense surface features (Talbot et al., 1993). Besides, the Class II hydrophobin of *M. grisea*, named Mhp1, is required for conidial development and viability and for surface hydrophobicity; indeed, the Δmhp1 mutant show a reduced appressorium formation and thus a significant reduction in pathogenicity (Kim et al., 2005). The *Botrytis cinerea* genome contains three genes encoding for HPs, one (Bhp1) belonging to Class I and two (Bhp2 and Bhp3) to Class II. While Izumitsu et al. (2010) attributed a role for Bhp1 in conidia hydrophobicity and adhesion to hydrophobic surfaces, Mosbach et al. (2011) showed that the *B. cinerea* HPs are neither involved in conferring surface hydrophobicity to conidia and aerial hyphae, nor they are required for virulence. Finally, Terhem and van Kan (2014) demonstrated that sclerotia produced by the double knock-out mutant ΔBhp1/ΔBhp3 and by the triple knock-out mutant were “easily wettable,” thus indicating that both Class I and Class II HPs are involved in normal development of *B. cinerea* apothecia. In *Aspergillus nidulans*, two HPs named RodA and DewA have been shown to contribute to conidiospore surface hydrophobicity (Grünbacher et al., 2014). Differently, the deletion of the hydrophobin encoding gene of the rye pathogen *Claviceps purpurea* (cpph1) did not lead to differences compared to wild type (Mey et al., 2003).

The genome of the plant pathogen *Fusarium graminearum*, a necrotrophic fungus causing the *Fusarium* head blight (FHB) disease of wheat, barley and other cereal grains, contains five different genes encoding for HPs, named FgHyd1-5. Four of them were predicted to belong to Class I HPs while the FgHyd5 seems to be the only gene coding for a Class II hydrophobin (Sarlin et al., 2012). So far, only the role of the FgHyd5 has been investigated, showing that it does not affect colony and hyphal morphology, it is not involved in the penetration of hyphae through the water-air interface but affects the hydrophobicity of aerial mycelia (Minenko et al., 2014).

Therefore, to fully characterize the role played by the five *F. graminearum* HPs in fungal growth and plant infection, single and triple mutants of the five genes have been produced and characterized *in vitro* and *in vivo*.

## Materials and Methods

### Sequences Analysis and Primer Design

The genome of *F. graminearum* (Wong et al., 2011; FGDB^1^) contains five genes encoding for Class I and Class II HPs (NCBI database entries: FGSG_01763: FgHyd1, FGSG_01764: FgHyd2, FGSG_09066: FgHyd3, FGSG_03960: FgHyd4 and FGSG_01831: FgHyd5) (Minenko et al., 2014). Prediction of signal peptides (SP) and hydrophobin conserved domains were performed by SMARTanalysis tool^2^ and Motif Scan (MyHits, SIB, Switzerland^3^), respectively. The five *F. graminearum* HPs were aligned and compared with the already characterized HPs of different Ascomycetes species using the ClustalW program, with the default settings for multiple alignments. A phylogenetic analysis was performed using BOOTSTRAP Neighbor Joining model TREE (1000) with MEGA 5 software.

The hydropathy score was calculated with ProtScale software^4^ by using the Kyle and Doolittle aa scale. All the primers used were designed by using PRIMER3^5^ and PerlPrimer v.1.1.17 software (Supplementary Table S1).

### *In vitro* Fungal Growth Conditions and Conidia Production

The fungal isolate *F. graminearum* wild type 8/1 (WT) was used to produce the mutants described in this study (Table 1). Macroconidia were obtained by culturing the fungal strains in carboxymethyl cellulose sodium salt (CMC; Sigma-Aldrich) as reported in Sella et al. (2016) or in liquid wheat media (WM). Liquid WM was prepared using 15 g of fresh wheat leaves blended in 1 L of distilled water, autoclaved twice and then filtrated. Three mL WM were inoculated with 10 μL of fungal conidia and incubated with 150 rpm shaking in the dark at 28°C for 5–7 days. Conidia were recovered by centrifugation at 4,000 rpm for 10 min at 4°C, and the pellet was re-suspended in 1 mL sterile deionised water to a final concentration of 10^6^ conidia mL^-1^. Macroconidia of all strains were stored as aqueous suspensions at -80°C. Conidiation assay was performed by inoculating 30 mL of CMC liquid medium or WM with 10^5^ conidia or 2 agar plugs (2-days old) of *F. graminearum* WT, single or triple mutants. Conidia were counted after 6 days of cultures with a haemocytometer using a bright light microscope (Zeiss, Axioscope). The experiments were performed by using two independent knock-out mutants for each gene obtaining similar results.

**Table 1:** *Fusarium graminearum* strains generated and used in this work.

Number	Strain	Resistance cassette	Reference
1	WT 8/1		Miedaner et al., 2000
2	WT 8/1 GFP constitutive	HygB	Jansen et al., 2005
**Single knock-out**			
3	Δ*Fghyd1* (FGSG_01763)	HygB	This study
4	Δ*Fghyd2* (FGSG_01764)	NptII	This study
5	Δ*Fghyd3* (FGSG_09066)	Nat1	This study
6	Δ*Fghyd4* (FGSG_03960)	HygB	This study
7	Δ*Fghyd5* (FGSG_01831)	HygB	This study
**Double knock-out**			
8	Δ*Fghyd23*	Nat1/NptII	This study
**Triple knock-out**			
11	Δ*Fghyd123*	Nat1/HygB/NptII	This study
12	Δ*Fghyd234*	Nat1/HygB/NptII	This study
13	Δ*Fghyd235*	Nat1/HygB/NptII	This study
**Single knock-out GFP constitutive**			
14	Δ*Fghyd1* GFP constitutive	NptII/HygB	This study
15	Δ*Fghyd2* GFP constitutive	NptII/HygB	This study
16	Δ*Fghyd3* GFP constitutive	Nat1/HygB	This study
17	Δ*Fghyd4* GFP constitutive	HygB/NptII	This study
18	Δ*Fghyd5* GFP constitutive	HygB/NptII	This study


*HygB, Hygromycin; Nat1, Nourseothricin acetyltransferase; NptII, Neomycin phosphotransferase.*

Colony morphology and aerial mycelia of the fungal strains were visually analyzed inoculating Complete Medium (CM) or Minimal Medium (MM) agar plates (85 mm diameter) prepared according to Leach et al. (1982) with a suspension of 10^5^ conidia mL^-1^ and incubating at 28°C.

*In vitro* growth was evaluated by inoculating CM and MM agar plates with 5-mm agar plugs of actively growing mycelia of WT and disruption mutants. Colony diameters were measured after 2 days of growth at 28°C in the dark. Two biological experiments each one consisting of 3 replicates per each strain were performed. The experiments were performed by using two independent knock-out mutants for each gene obtaining similar results. Data were statistically analyzed by applying the one way-Anova Bonferroni–Holm test.

### Generation of *F. graminearum* Hydrophobins Mutants

The deletion constructs *pALM-FgHyd1::Hyg, pALM-FgHyd2:: NptII, pJB-FgHyd3::Nat, pALM-FgHyd4::Hyg, pALM-FgHyd5:: Hyg* were generated using the yeast recombination method (Colot et al., 2006). Briefly, the 5′ and 3′ flanks of each gene were amplified with Dream-Taq polymerase (Thermo Scientific, Germany) using genomic DNA from the WT as template and oligonucleotides listed in Supplementary Table S1. Additionally, the resistance cassettes Nat, NptII or Hyg (Beck et al., 1982; Malonek et al., 2004; Maier et al., 2005) were amplified by PCR with Q5^^®^^ High-Fidelity DNA Polymerase (NEB) using the corresponding plasmids listed in Supplementary Table S2 as template and primers listed in Supplementary Table S1. All fragments, including the linearized pRS426 cloning vector, (Christianson et al., 1992) were gel-purified and subsequently used to transform the uracil auxotrophic yeast strain FGSC 9721 (FY 834) (Winston et al., 1995). The final constructs were excised with the restriction enzymes listed in Supplementary Table S2 and used to transform *F. graminearum* WT 8/1 strain. Protoplast preparation and fungal transformation were performed according to Maier et al. (2005). Disrupted mutants were generated by replacing the complete ORF of the gene of interest with the respective resistance cassette (Table 1). Selection of fungal transformed colonies was performed as reported by Quarantin et al. (2016) using the corresponding antibiotics. Resistant mutants were single-conidiated and screened by PCR using the primer pairs reported in Supplementary Table S1. The double mutant Δ*Fghyd23* was produced by transforming subsequently the single Δ*Fghyd3* mutant with the *pALM-Fghyd2::NptII* knock-out construct containing a different resistance cassette. Triple mutants were produced by transforming the double mutant Δ*Fghyd23* with the respective *pALM-Fghyd1::Hyg, pALM-Fghyd4::Hyg*, or *pALM-Fghyd5::Hyg* constructs. Transformants were then confirmed by Southern blot hybridization with probes obtained by PCR with primers shown in Supplementary Table S1 using digoxigenin labeled dUTP. Gel electrophoresis, restriction enzyme digestion, Southern blots and sequencing were performed using standard procedures. Hybridization was performed over-night at 68°C.

The generation of the single mutants constitutively expressing the green fluorescent protein (GFP) was performed as reported in Martínez-Rocha et al. (2016) by transforming each single deletion mutant with the constructs containing GFP-reporter gene (Lee et al., 2003; Supplementary Table S2).

### RNA Extraction and Expression Analysis by RT-qPCR

Expression analysis *in vitro* was performed using RNA extracted from *F. graminearum* WT and hydrophobin mutant mycelia grown on a cellophane layer placed on CM agarized medium at 28°C in the dark for 3 days. Expression analysis *in vivo* was performed using RNA extracted from six spikelets collected from the middle of wheat spikes inoculated with *F. graminearum* WT or hydrophobin mutants and incubated at 22°C for 5 days in a growth chamber with 16 h (hours) photoperiod. RNA was extracted with peqGOLD TriFast (PEQLAB Biotechnologie GmbH, Erlangen, Germany) according to the manufacturer’s instructions. Complementary DNA was prepared using the Revert-Aid H minus first-strand cDNA synthesis kit (Thermo Scientific, Germany). Primers used for expression analysis are listed in Supplementary Table S1. The expression level of hydrophobin genes was analyzed by RT-qPCR using the LightCycler 480 SYBR Green I Master mix (Roche Diagnostics GmbH, Mannheim, Germany) according to the manufacturer’s instructions. Relative gene expression was calculated using CP values of 3 technical replicates obtained from three independent biological samples. The housekeeping genes β-tubulin (FGSG_06611) and eIF5A (FGSG_01955) were used for normalization of gene-expression (Chen et al., 2011; Zheng et al., 2018). Both housekeeping genes showed equal expression stability under the given conditions (Supplementary Table S3), when compared using the BestKeeper expression tool (Pfaffl et al., 2004). The tool REST (Relative Expression Software Tool; Pfaffl et al., 2002) was used for relative expression analysis.

### Attachment to Hydrophobic Surface Assay

Six 50 μL sterile water drops containing 1,000 conidia of the *F. graminearum* WT or hydrophobin mutants were placed on a 85 mm Petri dish and incubated at 28°C in the dark. To maintain the humidity, a 35 mm diameter plate containing sterile water was placed inside the 85 mm Petri dish. Two Petri dishes containing six drops each were used for every strain (*n* = 12). After 24 h of germination, the drops were washed 3 times with sterile water and the remaining attached germlings were counted using a bright light microscope (Zeiss, Axioscope). The experiment was performed by using two independent knock-out mutants for each gene obtaining similar results.

### Penetration of Fungal Hyphae Through the Air-Liquid Barrier

The ability of the *F. graminearum* WT and mutant strains hyphae to penetrate through the water-air interface was tested by placing a 50 μL drop of CM liquid medium containing 500 conidia on 85 mm Petri dishes. Inside each plate, a smaller plate (35 mm diameter) containing water was inserted to maintain humidity condition. Three Petri dishes containing six drops each were used for every strain (*n* = 18). Plates were incubated at 28°C in the dark for up to 36–48 h until observing aerial hyphae growing out of the drops. Pictures were taken after 36 h with a stereo microscope (Leica ZFIII). Two biological experiments were performed by using two independent knock-out mutants for each gene obtaining similar results.

### Stress Response and Chitin Defect Assays

*Fusarium graminearum* WT and hydrophobin mutants were analyzed for ionic, osmotic and oxidative stress responses or for possible chitin defects at the cell wall by placing a 5 mm agar plugs containing 3 days old actively growing mycelia on CM plates containing 750 mM KCl, 1.5 M sorbitol, 30 mM hydrogen peroxide (H_2_O_2_) or 50 μg mL^-1^ calcofluor white (CFW). Sorbitol and KCl were added to the media before autoclaving. CFW and H_2_O_2_ were added after autoclaving and cooling down to 55°C. Plates were incubated at 28°C in the dark for 3 days. Colony diameters were measured every 24 h up to 3 days. Pictures were taken at 2 days. Each assay was performed with three replicates per each strain and the experiments were repeated at least three times. Data were statistically analyzed by applying the one way-Anova Bonferroni–Holm test.

### β-1,3-Glucanase and Chitinase Treatments

Agar plugs (7 mm diameter) containing actively growing mycelia of *F. graminearum* WT and hydrophobin mutants were inoculated on 10 mm diameter sterile paper disks imbibed with 1 U to 4 U of a β-1,3-glucanase enzymatic solution from *Trichoderma longibrachiatum* (Sigma-Aldrich) or with 1 U of *Trichoderma viride* chitinase enzymatic solution or liquid CM as negative control and placed on CM agar plates. The glucanase and chitinase enzymatic solutions were filtered with a 0.22 μm membrane filter prior to application. Plates were incubated in the dark at 28°C and fungal growth inhibition was measured after 48 h. Data of two independent experiments were statistically analyzed by applying the one way-Anova Bonferroni–Holm test.

### Tebuconazole Inhibition Assay

To investigate a possible alteration at the plasma membrane in the hydrophobin mutants, a 5 μL drop containing 500 conidia of the *F. graminearum* WT or hydrophobin mutant strains was added on CM agar plates supplemented with 0.01 μg mL^-1^ Tebuconazole (Folicur 250, Bayer), a systemic triazole fungicide that inhibits ergosterol biosynthesis. Plates were incubated in the dark at 28°C and the inhibition effect was determined by measuring the radial growth every 24 h up to 3 days and calculating the percentage of growth inhibition. Data of five independent experiments, each one including three replicates per strain, were statistically analyzed by applying the one way-Anova Bonferroni–Holm test.

### Point and Spray Inoculation Pathogenicity Assays on Wheat Plants

Wheat plants (*Triticum aestivum* cv. Nandu and Amaretto) were grown as reported in Paccanaro et al. (2017). Inoculation experiments were performed on wheat spikes at anthesis according to Woriedh et al. (2011). Briefly, point inoculations were performed by pipetting 10 μL of a fresh conidial suspension containing approximately 200 conidia between the palea and lemma of two opposite central spikelets. For spray inoculations, at first we determined the volume and how many conidia were dispersed per each spraying event. By using a spraying bottle containing a suspension of 10^6^ conidia mL^-1^ of WT or Δ*Fghyd3* mutant strain, affected in attachment and ability to penetrate the water-air interface, we calculated that each spray event approximately released 100 μL and 50,000 conidia for both strains. Spray inoculations were therefore performed by spraying conidia twice, one spray per each side of the spike. In both infection methods, after inoculation the spikes were covered with a plastic bag for 3 days to maintain a moist environment. Pictures and disease symptoms were assessed at 21 days post inoculation (dpi). Percentage of infection was determined by counting the number of visually diseased spikelets and relating to the total number of spikelets of the respective head. At least three independent infection experiments were performed by inoculating at least 10 spikes with each strain. Data were statistically analyzed by applying the one way-ANOVA Bonferroni–Holm test. The experiments were performed by using two independent knock-out mutants for each gene obtaining similar results.

### Infection Structures Production and Visualization by Fluorescence Microscopy

Wheat paleae (*T. aestivum* cv. Nandu), collected from spikes at anthesis, were washed once in 0.01% (v/v) Tween 20 for 10 min and twice in distilled sterile water. Eight paleae were laid on 1.6% (w/v) water agar plates and each one was inoculated with 10 μL of a fresh conidial suspension containing 2 × 10^4^ conidia mL^-1^ of *F. graminearum* WT strain or single hydrophobin mutants expressing GFP constitutively. Three plates were prepared for each strain. Plates were incubated on a chamber with a 16 h photoperiod at 22°C for 6 days. Infection structures produced were investigated by fluorescence microscopy as reported in Quarantin et al. (2016) with some modifications; in details, GFP was detected with an excitation of 488 nm and emission of 500–509 nm, while autofluorescence of the plant was excited at 405 nm and detected at 410–490 nm. Detection of infection structures was performed using the confocal Zeiss LSM 780 laser scanning microscope (LSM). Images were taken with Zeiss AxioCam MRm CCD camera. Generation of maximum intensity projections (MIP) of *z*-stacks were performed with Zeiss ZEN software (version 2010).

### Perithecia Formation and Ascospores Viability

Perithecia production and ascospores viability assays were performed according to Martínez-Rocha et al. (2016) with some changes. Briefly, five wheat stems, containing a node in the middle, were laid on water agar plate (1.6% w/v). Each node was inoculated with 10 μL conidial suspension containing 10^5^ conidia mL^-1^. Representative pictures of wheat nodes with perithecia were taken with a MZFLIII microscope (Leica Microsystems, Switzerland). Ascospores were re-suspended in 50 μL drop of CM and incubated for 4 h at 28°C in the dark. Microscopy pictures of germinated ascospores were taken with a Zeiss Axio Imager Z1 microscope. The assay was repeated three times with three replicates per strain by using two independent knock-out mutants for each gene obtaining similar results.

## Results

### Sequence Analysis and Phylogenetic Relationships of the *F. graminearum* HPs

**FIGURE 1:**
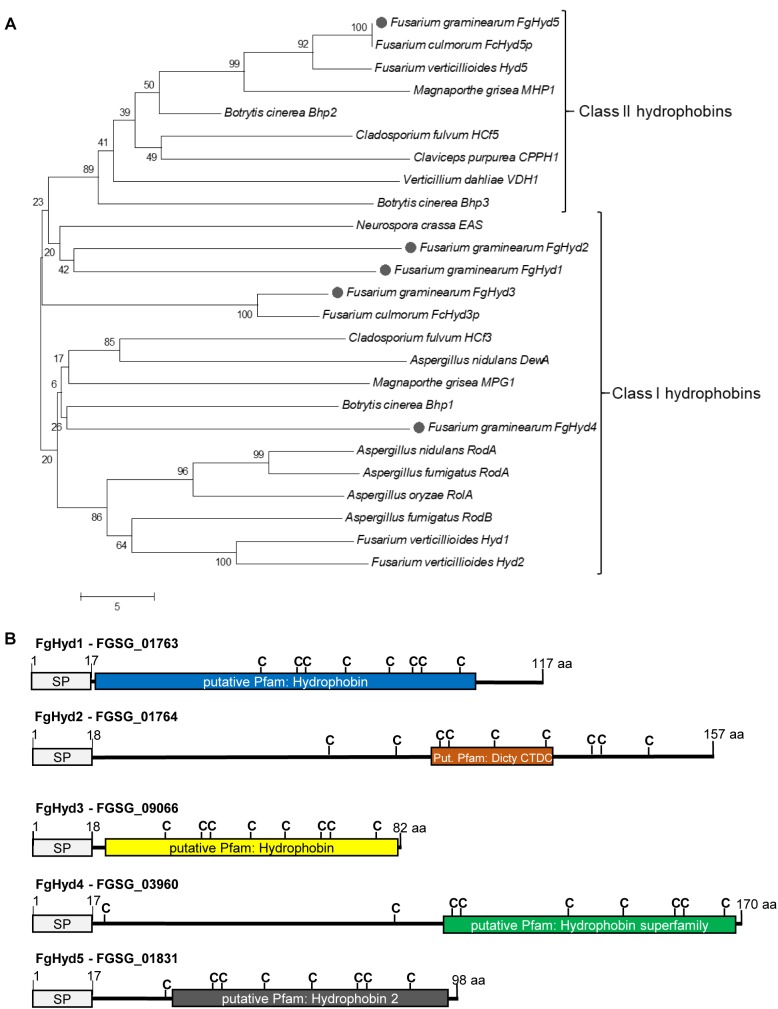
*Fusarium graminearum* hydrophobin family. Hydrophobin family of *F. graminearum* contains four class I (FgHyd1, FgHyd2, FgHyd3, and FgHyd4) and one class 2 (FgHyd5) hydrophobins. **(A)** Phylogenetic relationships of *F. graminearum* hydrophobins (gray circle) with hydrophobins from other ascomycetes demonstrate high homology among class 2, but low among class I hydrophobins. The Neighbor Joining model was built using MEGA 5 software. Robustness of the generated tree was determined using 1,000 bootstrap replicates. Bootstrap values are provided at the beginning of each branch and are given as a percentage. The fungal proteins used are: *Cladosporium fulvum* HCf3 (CAD92803); *C. fulvum* HCf5 (CAC27408); *F. graminearum* FgHyd1 (FGSG_01763); *F. graminearum* FgHyd2 (FGSG_01764); *F. graminearum* FgHyd3 (FGSG_09066); *F. graminearum* FgHyd4 (FGSG_03960); *F. graminearum* FgHyd5 (FGSG_01831); *Fusarium verticillioides* Hyd1 (Q6YF32); *F. verticillioides* Hyd2 (Q6YF31); *F. verticillioides* Hyd5 (Q6YD93); *Botrytis cinerea* Bhp1 (BC1G_15273); *B. cinerea* Bhp2 (BC1G_03994); *B. cinerea* Bhp3 (BC1G_01012); *Aspergillus oryzae* RolA (BAC65230.1); *Verticillium dahliae* VDH1 (AAY89101.1); *Magnaporthe grisea* MPG1 (P52751); *M. grisea* MHP1 (AAD18059); *Fusarium culmorum* FcHyd3p (ABE27987.1); *F. culmorum* FcHyd5p (ABE27986.1); *Neurospora crassa* EAS (EAA34064.1); *Claviceps purpurea* CPPH1 (CAD10781.1); *Aspergillus nidulans* RodA (AAA33321.1); *A. nidulans* DewA (AAC13762.1); *Aspergillus fumigatus* RodA (AAB60712.1) and *A. fumigatus* RodB (EAL91055.1). The corresponding accession numbers were obtained from the NCBI database (http://www.ncbi.nlm.nih.gov/). **(B)** Schematic representation of *F. graminearum* hydrophobin proteins displaying classical hydrophobin characteristics, signal peptide for secretion (SP, signal peptide), multiple cysteins (C, cystein) and low number of amino acids (aa, small proteins). Prediction of SP was performed using SignalP. Prediction of hydrophobin domains was performed using Motif Scan (MyHits, SIB, Switzerland). Dicty_CTDC *Dictyostelium* (*slime mold*) *repeat.*

The five hydrophobin encoding genes present in the *F. graminearum* genome (FgHyd1: FGSG_01763; FgHyd2: FGSG_01764; FgHyd3: FGSG_09066; FgHyd4: FGSG_03960 and FgHyd5: FGSG_01831) show similarity with known hydrophobin sequences. Phylogenetic analysis of *F. graminearum* and other known Ascomycetes HPs reveals the separation of the proteins into two clades that roughly correspond to the two defined Classes of HPs. As expected (Minenko et al., 2014), FgHyd1, FgHyd2, FgHyd3, and FgHyd4 belong to the clade of the Class I HPs and FgHyd5 to that of the Class II (Figure 1A). Furthermore, phylogenetic relationships demonstrate high similarity among Class II hydrophobins and low similarity among those of Class I (Figure 1A and Supplementary Figure S1). Their amino acid (aa) sequences present HPs defining characteristics such as secretion signal peptide, multiple cysteines in a conserved array and small protein size from 82 to 170 aa (Figure 1B and Supplementary Figure S1). The predicted hydrophobin domain has been identified in four of the proteins, while the FgHyd2 shows a *Dictyostelium* repeat as putative Pfam domain (Figure 1B). Hydropathy profiles validate the hydrophobic properties of *F. graminearum* HPs with FgHyd3, FgHyd4, and FgHyd5 presenting the higher number of hydrophobic residues and higher hydropathy scores (Supplementary Figure S2).

### The *F. graminearum* FgHyd3 Gene Is Highly Up-Regulated During Fungal Growth and Wheat Infection

**FIGURE 2:**
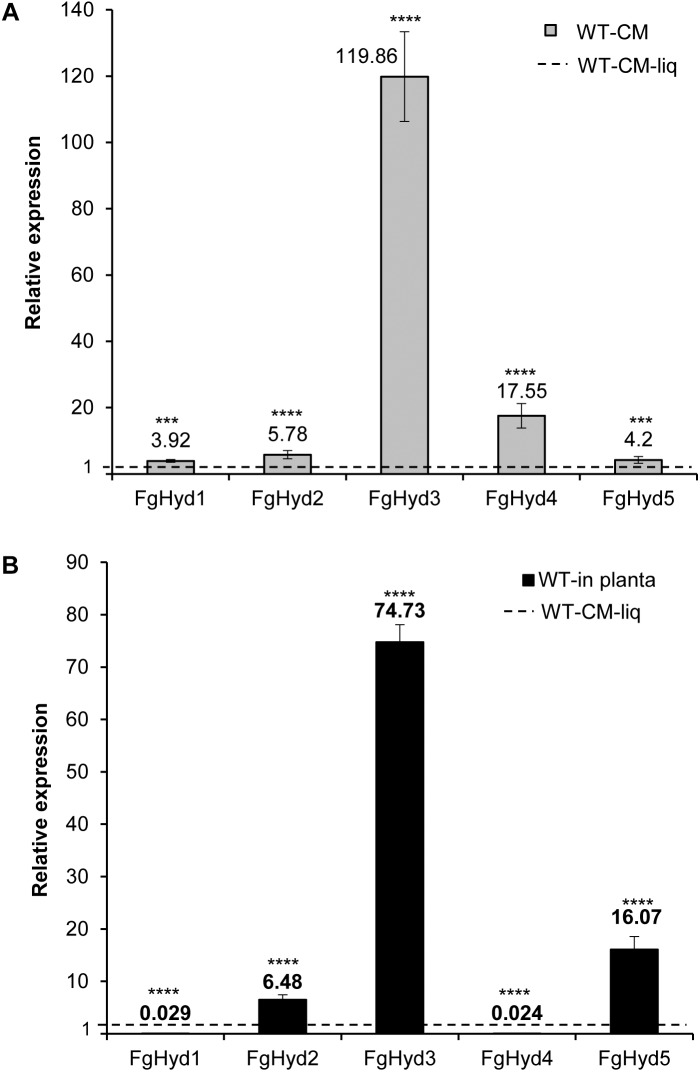
FgHyd3 gene is the most highly expressed hydrophobin in *F. graminearum*. Expression analysis of hydrophobin genes by RT-qPCR demonstrated that Fghyd3 hydrophobin gene is the most highly expressed when *F. graminearum* wild type strain (WT) grows on an artificial hydrophobic surface or during infection of wheat spikes. **(A)** RNA extracted from mycelia of WT grown on agarized complete media (CM) with a cellophane layer for 3 days at 28°C in the dark, and WT grown in liquid CM media (CM-liq) for 3 days at 28°C with 150 rpm were used to quantify the relative expression of hydrophobin genes in axenic culture. **(B)** RNA extracted from wheat spikes inoculated with 200 conidia of WT (WT-in planta) for 5 days at 22°C with a photoperiod of 16/8 h light was used to quantify the relative expression of hydrophobin genes during infection. b-tubulin and EIF-5a (eukaryotic translation initiation factor 5A) were used as normalizers and expression on WT grown in liquid as calibrator (set to 1). Error bars indicate standard deviation calculated from data per triplicate (3 biological samples and 3 experimental replicates). Statistical analysis was calculated with respect to the WT using one way-Anova Bonferroni–Holm (significance: ^∗∗∗∗^*p* < 0.0001, ^∗∗∗^*p* < 0.001).

To investigate the expression levels of hydrophobin genes during fungal growth and wheat spike infection, we extracted RNA from *F. graminearum* WT mycelium grown in liquid CM or on a cellophane layer placed on the surface of an agarized CM culture, and from inoculated wheat heads. Compared to their expression in CM liquid culture, all hydrophobin genes were over-expressed during fungal growth on the artificial hydrophobic cellophane layer. In particular, FgHyd3 gene was the most highly expressed, followed by FgHyd4, FgHyd2, FgHyd5, and FgHyd1 (Figure 2A). In contrast, not all hydrophobin genes were highly expressed *in planta*. The expression analysis demonstrated that FgHyd3 was the most highly expressed followed by FgHyd5 and FgHyd2, while FgHyd1 and FgHyd4 were faintly expressed (Figure 2B).

### Production of Single and Triple Disruption Mutants of *F. graminearum* Hydrophobin Genes

To establish the individual and combined role of HPs on *F. graminearum* growth *in vitro* and *in vivo*, we generated disruption mutants of the five *F. graminearum* hydrophobin genes by targeted homologous recombination and the obtained single mutants were analyzed by PCR and then by Southern blot confirming the deletion of the genes of interest (Supplementary Figures S3–S7). We also generated the triple knock-out mutants Δ*Fghyd123*, Δ*Fghyd234*, and Δ*Fghyd235* by combining the constructs with different antibiotic resistance and confirmed gene disruption as reported above (Supplementary Figures S3–S7).

### Deletion of the FgHyd2 Gene Reduces Mycelia Growth

**FIGURE 3:**
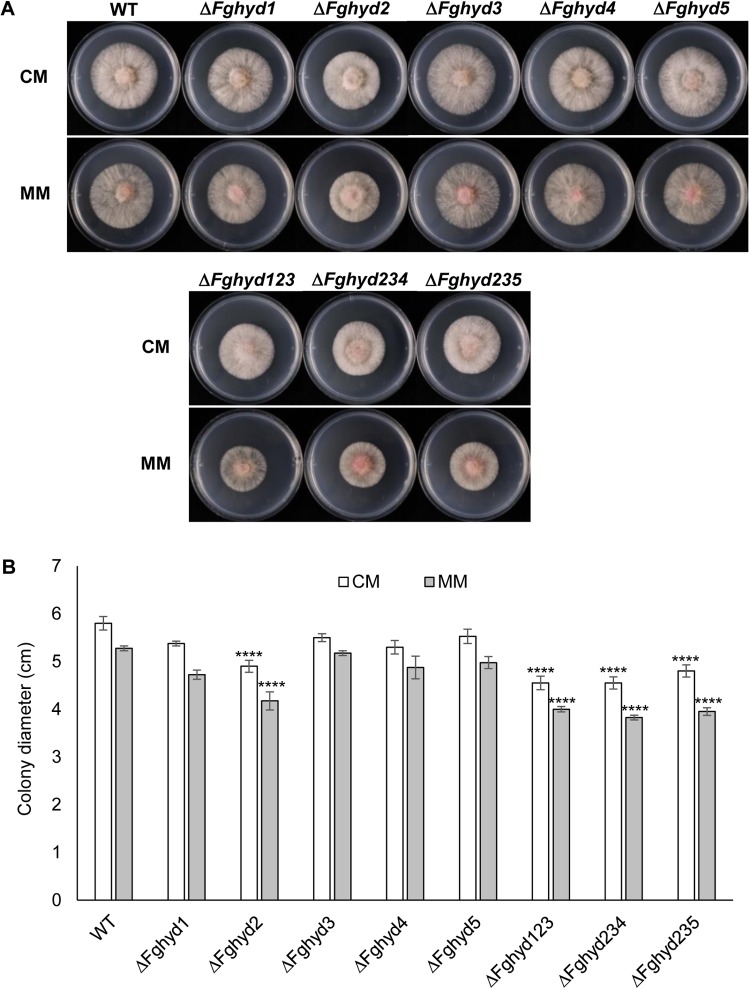
*Fusarium graminearum* hydrophobins deletion mutants phenotype on complete or minimal media. 5 mm agar blocks of actively growing deletion mutants or WT were placed on CM or minimal media (MM) and grown for 2 days at 28°C in the dark. Hydrophobin deletion mutants grew similarly to the WT with exception of Δ*Fghyd2* and triple mutants which grew slower. **(A)** Pictures of WT and hydrophobins deletion mutants were taken at 2 dpi. **(B)** Graph showing the average of colonies diameter. Error bars indicate standard deviation calculated from data representative of 2 biological experiments and 3 replicates. Statistical analysis of each treatment was calculated with respect to the WT using one way-Anova Bonferroni–Holm (significance: ^∗∗∗∗^*p* < 0.0001). The experiment was performed by using two independent knock-out mutants for each gene obtaining similar results.

To investigate the effect of the deletion of the hydrophobin genes on *F. graminearum* growth, the WT strain and the deletion mutants were grown on CM or MM for 4 days. No significant difference in the visual appearance of mycelia was observed; mutants retained the ability to form radial colonies and the mycelia appeared white with the production of aerial hyphae (Figure 3A). Interestingly, the Δ*Fghyd2* single mutant and all the three triple mutants presented a reduced radial growth compared to the WT and the other single mutants. Since this phenotype was similar in both CM and MM, all the subsequent analyses were performed on CM (Figure 3B). No significant differences were observed between the WT and all the mutant strains in the number of conidia produced in CMC medium (Supplementary Figure S8) and the same result was obtained on liquid WM.

### Mutants Without the FgHyd2 Gene Grow as WT Under Stress Conditions

To determine the importance of the HPs in response to ionic, osmotic or oxidative stresses, we compared the growth rate of mutants and WT on agarized CM supplemented with 750 mM KCl, 1.5 M sorbitol, or 30 mM H_2_O_2_, respectively.

**FIGURE 4:**
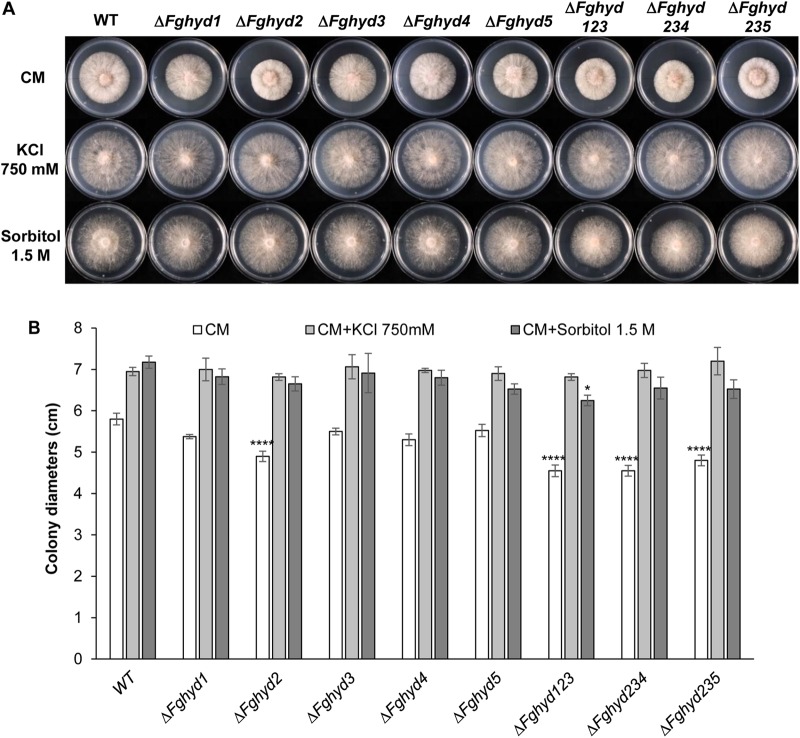
Ionic and osmotic stress response of *F. graminearum* hydrophobin mutants on complete and minimal medium. Plates containing CM or CM supplemented with 750 mM KCl or 1.5 M sorbitol were inoculated with 5 mm plugs of actively growing mycelia of the WT strain, single or triple deletion mutants. Plates were incubated at 28°C in the dark. **(A)** Pictures representative of each treatment were taken at 2 dpi. **(B)** The susceptibility to stress was estimated by measuring colony diameters at 2 dpi. Error bars indicate standard deviation calculated from data representative of 2 biological experiments and 3 experimental replicates. Statistical analysis of each treatment was calculated with respect to the WT using one way-Anova Bonferroni–Holm (significance: ^∗^*p* < 0.05, ^∗∗∗∗^*p* < 0.0001).

**FIGURE 5:**
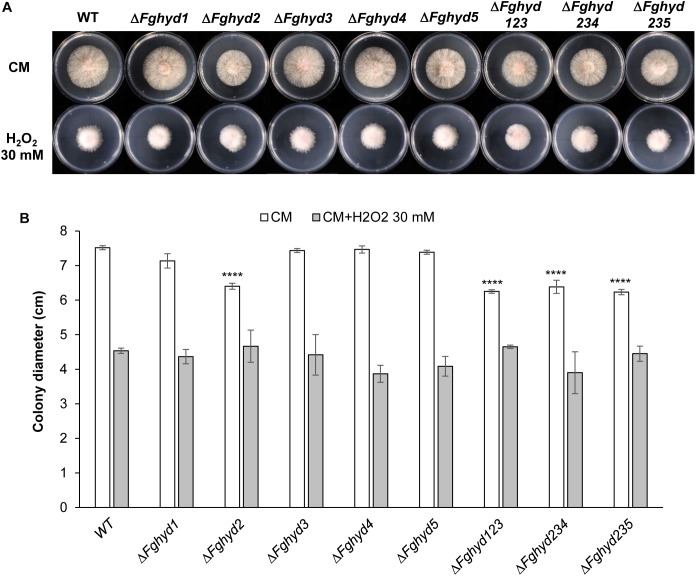
Oxidative stress response of *F. graminearum* hydrophobin mutants on complete and minimal medium. Plates containing CM or CM supplemented with 30 mM H_2_O_2_ were inoculated with 5 mm plugs of actively growing mycelia of the WT strain, single or triple deletion mutants. Plates were incubated at 28°C in the dark. **(A)** Pictures representative of each treatment were taken at 2 dpi. **(B)** The susceptibility to stress was estimated by measuring mycelia diameters after 2 days. Error bars indicate standard deviation calculated from data representative of 2 biological experiments and 3 experimental replicates. Statistical analysis of each treatment was calculated with respect to the WT using one way-Anova Bonferroni–Holm (significance: ^∗∗∗∗^*p* < 0.0001).

Interestingly, the colony diameters of all the strains under ionic and osmotic stress were higher compared to their growth on CM (Figure 4A,B), although the mycelium was rather sparse (Figure 4A), thus indicating a certain level of stress, while the colony diameters were negatively affected by the oxidative stress (Figure 5A,B). Under all the stress conditions tested, the mutants’ growth did not present significant differences compared to the WT and the growth defect of the single Δ*Fghyd2* and triple mutants observed on CM was remediated (Figure 4A, 5A).

### *F. graminearum* Hydrophobin Mutants Do Not Show Alterations at the Cell Wall

To test possible alterations at the cell wall, we compared the growth of mutants and WT on agarized CM supplemented with calcofluor white (CFW, 50 μg mL^-1^), a compound able to prevent the *in vivo* assembly of chitin (Roncero and Durán, 1985), or after a treatment with the fungal cell wall degrading enzymes β-1,3-glucanase or chitinase.

All the colonies were restricted by CFW without significant differences between the WT and the mutants (Supplementary Figures S9A,B).

The β-1,3-glucanase treatment did not affect the growth of WT and hydrophobin mutants, even when the dose of the enzyme was increased up to 4 U (Supplementary Figure S10). Differently, 1 U of chitinase clearly inhibited the fungal growth, although no significant differences were observed among WT and mutants (Supplementary Figure S10).

### *F. graminearum* Hydrophobin Triple Mutants Exhibit Higher Susceptibility to Tebuconazole Fungicide

**FIGURE 6:**
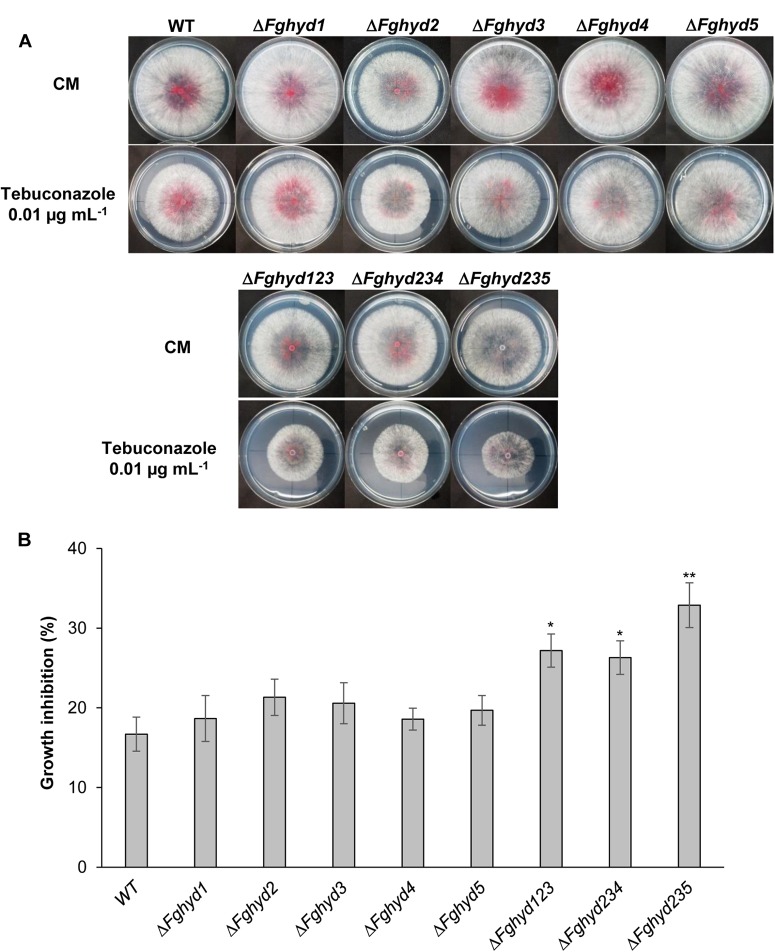
Triple hydrophobin mutants are more sensitive to the fungicide tebuconazole. While the single mutants grew similarly to the WT, the triple deletion mutants grew slower on minimal media supplemented with 0.01 μg mL^-1^ tebuconazole. **(A)** Plates with CM or CM supplemented with the fungicide tebuconazole (Folicur 250, Bayer) were inoculated with 5 μL of a conidial suspension of 1 × 10^5^ conidia mL^-1^ of the WT strain, single or triple hydrophobin mutants. Plates were incubated in the dark at 28°C. Pictures were taken at 3 dpi. **(B)** Percentage of growth inhibition was determined by measuring the radial growth at 3 dpi. Data represent the mean ± standard error (indicated by bars) of five independent experiments. Statistical analysis was calculated with respect to the WT using one way-Anova Bonferroni–Holm (significance: ^∗^*p* < 0.05, ^∗∗^*p* < 0.01).

The effect of the fungicide tebuconazole, an ergosterol biosynthesis inhibitor, on *F. graminearum* hydrophobin mutants was also determined by measuring the radial growth after 4 days. Representative pictures are reported in Figure 6A. All the single mutants, in particular Δ*Fghyd2* and Δ*Fghyd3*, appeared slightly but not significantly more inhibited compared to WT, while the triple mutants showed a higher and significantly different inhibition percentage compared to WT (Figure 6B). In particular, the Δ*Fghyd235* mutant was significantly more inhibited by about 50% compared to the WT strain (Figure 6B).

### Deletion of FgHyd3 Affects the Ability of Fungal Germlings to Attach to Hydrophobic Surface

**FIGURE 7:**
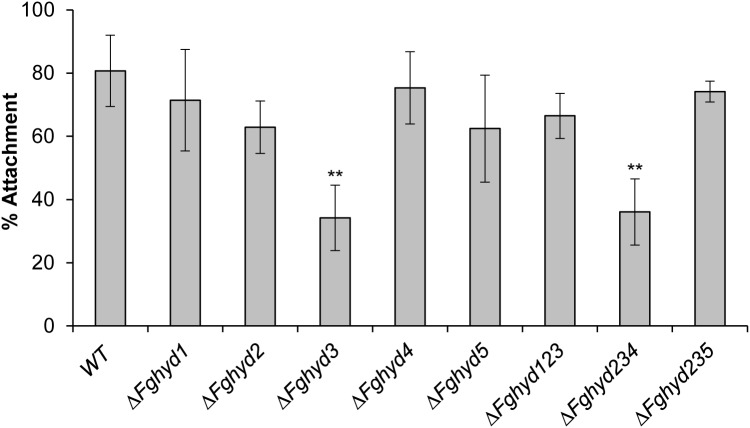
FgHyd3 is necessary for hydrophobic surface attachment. Six drops containing 50 μL sterile water and 1,000 conidia each were placed on the inner surface of Petri dishes and incubated at 28°C on the dark. After 24 h, fungal germlings were washed 3 times and counted. 1,000 conidia counted represent 100% of attachment. Error bars indicate standard deviation calculated from data representative of 2 biological experiments and 6 replicates each. Statistical analysis was calculated with respect to the WT using one way-Anova Bonferroni–Holm (significance: ^∗∗^*p* < 0.01). Only Δ*Fghyd*3 and triple Δ*Fghyd*234 mutants have a defect in attachment to hydrophobic surfaces. The experiment was performed by using two independent knock-out mutants for each gene obtaining similar results.

To evaluate the ability of fungal germlings to attach to a hydrophobic surface, we placed water drops containing about 1,000 conidia on the inner surface of Petri dishes and, after 24 h of incubation, the drops were washed off with water and the number of fungal germlings still attached to the surface was counted by a bright field microscope. The Δ*Fghyd3* mutant as well as the triple mutant Δ*Fghyd234* were significantly impaired in attachment in comparison to WT, while the other mutants were slightly but not significantly impaired in attachment (Figure 7).

### Deletion of FgHyd2 and FgHyd3 Affects Hyphal Ability to Penetrate the Water-Air Interface

**FIGURE 8:**
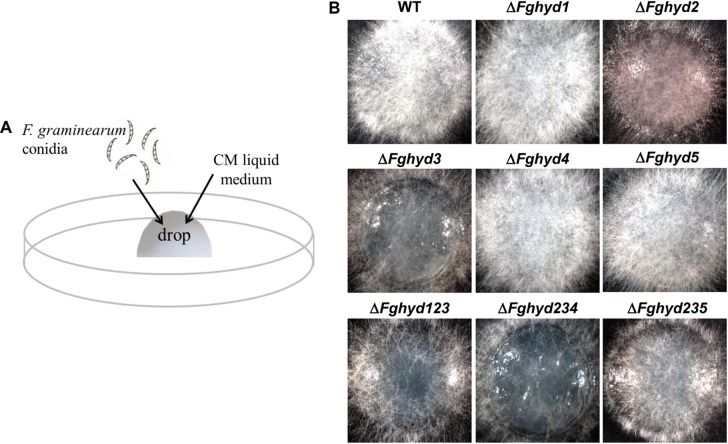
FgHyd2 and FgHyd3 are necessary for breaking the liquid-air interface. **(A)** A 50 μL drop of CM containing 500 conidia of the WT strain or hydrophobin deletion mutants were placed on the inner surface of Petri dishes and allowed to grow for 36 h at 28°C in the dark. **(B)** Pictures were taken with the stereo microscope (Leica- ZDFIII) at 36 h. Hyphae of single deletion mutants Δ*Fghyd2*, Δ*Fghyd3* and triple deletion mutant Δ*Fghyd*234 were not able to break the liquid-air interface. Triple deletion mutants Δ*Fghyd1*23 and Δ*Fghyd*235 displayed less mycelia passing through the liquid-air interface than the WT. The experiment was performed by using two independent knock-out mutants for each gene obtaining similar results.

To evaluate the hyphal ability to penetrate the water-air interface, we performed a drop assay using liquid CM (Figure 8A). Δ*Fghyd2*, Δ*Fghyd3* and the triple mutants, especially Δ*Fghyd234*, exhibited a reduction in the number of aerial hyphae passing through the air-liquid barrier, thus indicating a lower ability of the hyphae to overcome the water-air interface and a possible contribution of FgHyd2 and FgHyd3 to the surface hydrophobicity of the *F. graminearum* aerial mycelia (Figure 8B).

### Expression Analysis of Hydrophobin Genes in the Hydrophobin Mutants Grown on Artificial Hydrophobic Surface

**FIGURE 9:**
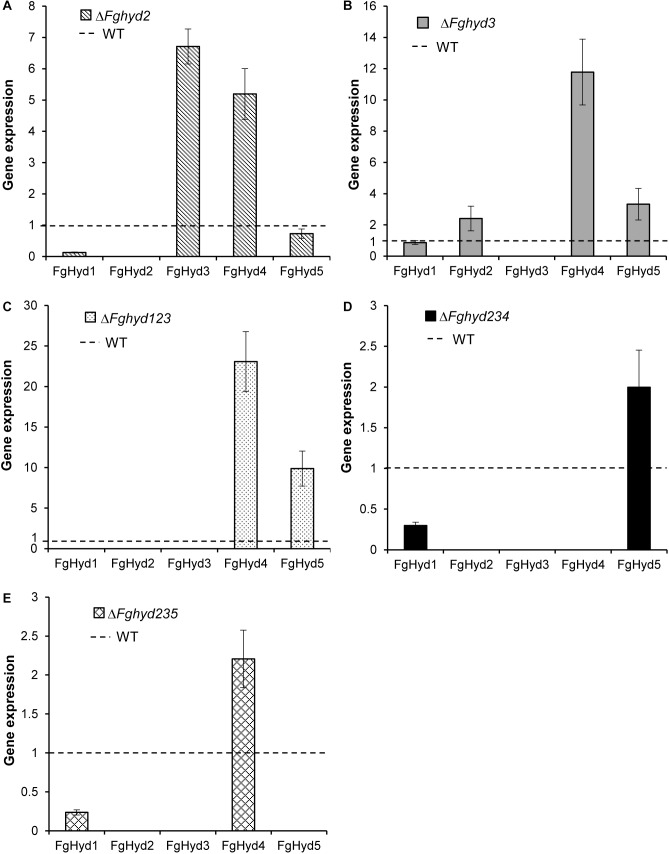
Hydrophobin genes expression in hydrophobin deletion mutants grown on artificial surface. RNAs extracted from mycelia of WT strain, Δ*Fghyd2*, Δ*Fghyd3* and triple mutants grown on agarized CM with a cellophane layer were extracted and used for expression analysis using RT-qPCR. b-tubulin and EIF-5a were used as normalizers and expression on WT under similar conditions as calibrator (set to 1). **(A)** Lack of FgHyd2 gene increased expression of FgHyd3 and FgHyd4. **(B)** FgHyd2, FgHyd4, and FgHyd5 were up-regulated on the Δ*Fghyd3* mutant. **(C)** FgHyd4 and FgHyd5 were strongly expressed on the triple mutant Δ*Fghyd123* compared to WT. **(D)** Expression of FgHyd5 was moderately increased on the triple deletion mutant Δ*Fghyd234*. **(E)** FgHyd4 expression was increased on the triple deletion mutant Δ*Fghyd235*. Error bars indicate standard error calculated from data per triplicate using the relative expression analysis tool REST (Relative Expression Software Tool).

The expression of the hydrophobin genes was analyzed on the Δ*Fghyd2* and Δ*Fghyd3* single mutants as well as on the triple mutants compared to WT. To this aim, strains were grown on a cellophane layer placed on agarized CM. The FgHyd1 gene was down-regulated in all the hydrophobin mutants except the Δ*Fghyd3* mutant (Figure 9). The disruption of the FgHyd2 gene increased the expression of FgHyd3 and FgHyd4 genes, while the deletion of FgHyd3 increased the expression of FgHyd2, FgHyd5 and mostly FgHyd4 genes (Figure 9A,B). In the triple hydrophobin mutant Δ*Fghyd123*, FgHyd4 and FgHyd5 were strongly up-regulated (Figure 9C). The expression of the FgHyd5 or FgHyd4 genes was moderately up-regulated in the triple deletion mutants Δ*Fghyd234* and Δ*Fghyd235*, respectively (Figure 9D,E).

### FgHyd2, FgHyd3, and FgHyd4 Are Involved in Adhesion of Conidia to Wheat Spikes During the Early Stages of the Infection Process

**FIGURE 10:**
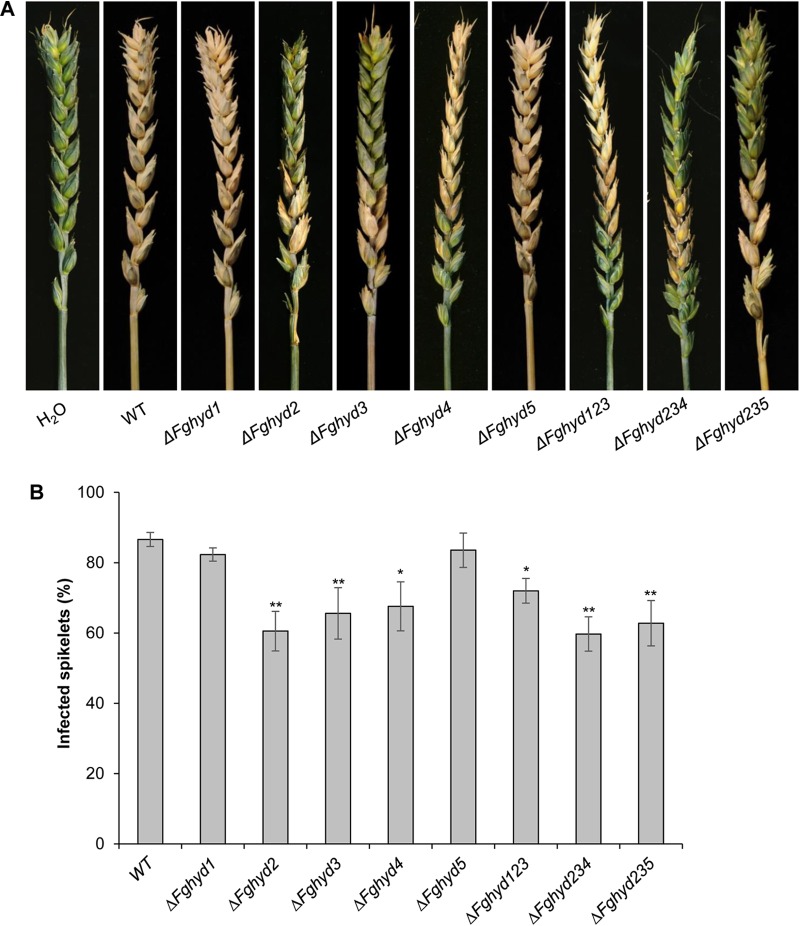
Hydrophobins of *F. graminearum* are necessary for virulence. **(A)** Wheat spikes of the susceptible cultivar Nandu were sprayed twice with 100 μL of a conidial suspension containing 500 conidia μL^-1^ of the WT strain, single and triple mutants. After 24 h the spikes were sprayed twice with 2 mL of water. The spikes sprayed with the single mutants Δ*FgHyd2*, Δ*FgHyd3*, and Δ*FgHyd4* as well as the triple mutants developed less symptoms than the WT, while Δ*FgHyd1* and Δ*FgHyd5* mutants developed similar symptoms than the WT. Water was used as mock inoculation. **(B)** Disease symptoms were assessed at 21 dpi by counting the number of visually diseased spikelets on wheat cultivar Nandu. Infected spikelets are expressed as percentage of symptomatic spikelets on total number of spikelets of the respective head. Data represent the mean ± standard error (indicated by bars) of 10 independent infection experiments. Statistical analysis was calculated with respect to the WT using one way-Anova Bonferroni–Holm (significance: ^∗^*p* < 0.05, ^∗∗^*p* < 0.01). The experiment was performed by using two independent knock-out mutants for each gene obtaining similar results.

To determine whether the *F. graminearum* HPs are involved in pathogenicity, point inoculations of wheat spikes (cv. Nandu) with the WT, single and triple mutant strains were performed. At 21 dpi all the mutants showed full virulence compared to the WT strain (Supplementary Figure S11). To better clarify the role of *F. graminearum* HPs in the adhesion of conidia to wheat spikes and in order to reproduce natural infection conditions, we also performed the inoculation by spraying the spores of WT and mutants on flowering wheat spikes (cv. Nandu). Representative pictures of infected spikes at 21 dpi are reported in Figure 10A. While spikes inoculated with the Δ*Fghyd1* and Δ*Fghyd5* mutants presented a similar percentage of infected spikelets compared to WT, spikes inoculated with Δ*Fghyd2*, Δ*Fghyd3*, and Δ*Fghyd4* mutants exhibited a significant reduction of infection symptoms by about 30, 25, and 20%, respectively, at 21 dpi (Figure 10B). Spray inoculations performed with the triple mutants showed also a significant reduction in the percentage of infected spikelets by about 30, 25, and 20% for Δ*Fghyd235*, Δ*Fghyd234*, and Δ*Fghyd123*, respectively (Figure 10B).

In addition, we tested the ability of the two single mutants showing higher reduction in the percentage of infected spikelets (Δ*Fghyd2* and Δ*Fghyd3*) to infect a different wheat cultivar (cv. Amaretto) with increased resistance to *F. graminearum* infections. Spray inoculations produced results similar to those obtained on cv. Nandu (Supplementary Figures S12A–C). In particular, at 21 dpi cv. Amaretto spikes inoculated with Δ*Fghyd2* and Δ*Fghyd3* mutants showed a reduction in symptomatic spikelets by about 35 and 25%, respectively (Supplementary Figures S12B–D). Taken together, spray inoculation results indicate that FgHyd4 and mostly FgHyd2 and FgHyd3 are involved in adhesion of conidia to the wheat spike surface.

### Expression Analysis of Hydrophobin Genes in the Hydrophobin Mutants During Wheat Infection

**FIGURE 11:**
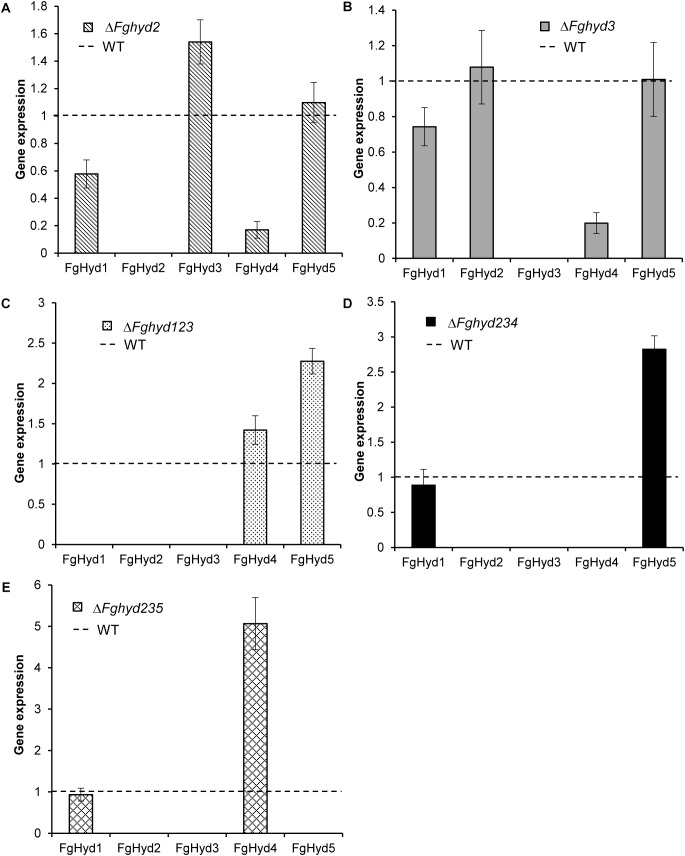
Expression analysis of hydrophobin genes during infection. RNAs extracted from wheat spikes inoculated with 10 μL of a conidial suspension of 2 × 10^4^ conidia mL^-1^ of WT strain, Δ*Fghyd2*, Δ*Fghyd3* and triple mutants were extracted at 5 dpi and used for expression analysis using RT-qPCR. b-tubulin and EIF-5a were used as normalizers and expression on WT under similar conditions as calibrator (set to 1). **(A)** FgHyd3 gene expression increased and expression of FgHyd1 and FgHyd4 decreased in Δ*Fghyd2* mutant. **(B)** FgHyd4 gene expression decreased on the Δ*Fghyd3* mutant, while other hydrophobin genes had no change in expression. **(C)** FgHyd4 and FgHyd5 had higher expression on the triple mutant Δ*Fghyd123* compared to WT. **(D)** Expression of FgHyd5 increased on the triple deletion mutant Δ*Fghyd234*. **(E)** FgHyd4 expression was highly increased on the triple deletion mutant Δ*Fghyd235* compared to WT. Error bars indicate standard error calculated from data per triplicate using the relative expression analysis tool REST (Relative Expression Software Tool).

To investigate the expression during wheat infection of the hydrophobin genes in the Δ*Fghyd2* and Δ*Fghyd3* single mutants and in the triple mutants compared to WT, we extracted RNA from spikes at 5 dpi. Results showed that disruption of the FgHyd2 gene slightly increased the expression of FgHyd3, while expression of FgHyd1 and FgHyd4 was down-regulated (Figure 11A). On the other hand, the deletion of the FgHyd3 gene decreased the expression of FgHyd4, while the expression of the other hydrophobin genes showed no significant change (Figure 11B). In the triple mutant Δ*Fghyd123*, the expression of the FgHyd4 and mostly FgHyd5 genes was higher compared to WT (Figure 11C). The expression of the FgHyd5 or FgHyd4 genes was up-regulated in the triple deletion mutants Δ*Fghyd234* and Δ*Fghyd235*, respectively, while the expression of the FgHyd1 gene was similar to WT in both triple mutants (Figure 11D,E).

### *F. graminearum* HPs Are Not Required for Infection Structures Formation or Sexual Reproduction

To determine whether the hydrophobin genes are required for infection structure formation, we performed a histological analysis by inoculating wheat paleae with a spore suspension of WT or single mutant strains constitutively expressing GFP. Fluorescence microscopy showed that the hydrophobin mutants produced normal infection structures, such as infection cushions, similar to WT (Supplementary Figure S13).

In addition, the *F. graminearum* WT, single and triple mutant strains were tested for perithecia production on wheat nodes in order to better reproduce natural conditions. After 35 days, all the mutant strains confirmed the ability to develop normal perithecia (Supplementary Figure S14A). Moreover, germination of ascospores from perithecia was assessed using bright field microscopy. All ascospores germinated after 4 h of incubation in liquid CM (Supplementary Figure S14B), thus indicating that the *F. graminearum* HPs are not essential for sexual reproduction or ascospore germination of the fungus.

## Discussion

Hydrophobins are small cysteine-rich surface-active proteins that are secreted only by fungi and may play several roles during fungal growth and plant infection (Wessels, 2000; Wösten, 2001; Whiteford and Spanu, 2002). Indeed, HPs contribute to aerial structures escaping the aqueous environment, to sensing of external surface features, to the attachment of hyphae and spores to hydrophobic surfaces, to the development of pre-infection structures and to virulence (Wessels, 2000; Wösten, 2001; Whiteford and Spanu, 2002).

This research focuses on the characterization of single and triple knock-out mutants of the five *F. graminearum* hydrophobin genes (FgHyd1-5) in order to understand the contribution of the corresponding HPs to mycelium growth in agar-solidified substrates supplemented with ionic, osmotic or oxidative stressors and to hyphal protection against harmful compounds. Further important functions of HPs investigated in the *F. graminearum* mutants are hyphal penetration at water-air interface, attachment of fungal germlings to hydrophobic surface, virulence on wheat spikes and formation of reproductive structures on wheat surface. Besides, the expression of the FgHyd genes on hydrophobic surfaces and during spike infection has been analyzed together with possible effects of gene disruption on the transcription of the non-deleted FgHyd genes.

On agar substrates, none of the mutants presented an altered morphology of colonies, but the Δ*Fghyd2* and the triple mutants with the FgHyd2 deleted exhibited a reduced radial growth. This result suggests a role of the FgHyd2 in proper hyphal growth. In fact, it is known that HPs not only provide spores and hyphae with a hydrophobic coat, but also affect the cell wall architecture (van Wetter et al., 2000). However, the deletion of the FgHyd genes does not seem to cause noticeable cell wall defects, since, compared to WT, all the Δ*Fghyd* mutants are not differently affected by calcofluor white, a compound able to prevent the *in vivo* assembly of chitin, and by the cell wall degrading enzymes chitinase and β-1,3-glucanase. This feature functionally distinguishes the FgHyds from the *F. graminearum* cerato-platanins (FgCPPs), another class of small secreted non-catalytic cysteine-rich proteins, which instead play a protective role against the cell wall degrading enzymes (Quarantin et al., 2016).

Interestingly, the Δ*Fghyd2* and the triple mutants showing growth defect resumed growth levels comparable to that of WT under ionic, osmotic and oxidative stress conditions. A similar behavior has been previously observed on yeast cultures. Indeed, the ability to withstand several stresses, such as oxidative, acid or heat stress, was increased on slow-growing yeast mutants, strongly suggesting an inverse correlation existing between mutant’s growth rate and stress tolerance (Zakrzewska et al., 2011). Nevertheless, the biological function of this mechanism in *F. graminearum* remains to be elucidated.

Possible defects of mutants were also investigated growing them in the presence of tebuconazole, a systemic triazole fungicide containing an ergosterol biosynthesis inhibitor. While the single mutants appeared slightly but not significantly inhibited by this treatment, the triple mutants showed a significantly higher susceptibility compared to WT. To date, a protecting role of HPs for aerial conidia and hyphae has been suggested only against desiccation and wetting (Wösten, 2001; Whiteford and Spanu, 2002; Klimes and Dobinson, 2006). Our result suggests that the simultaneous presence of different HPs on the conidia and hyphal surfaces could form a protecting shield against toxic compounds, although future studies are needed to clearly demonstrate the localization of *F. graminearum* HPs.

The aerial hyphae of Δ*Fghyd2* and Δ*Fghyd3* single mutants, as well as the triple mutants with disrupted FgHyd2 and FgHyd3 genes, especially the Δ*Fghyd234* one, showed a strongly impaired ability to penetrate through the water-air interface. Indeed, by self-assembling at the water-air interface and forming a hydrophobic layer, HPs are known to play a crucial role at the surface of fungal hyphae, conferring water-repellent properties to escape the aqueous environment (Wösten et al., 1999; Wösten and Scholtmeijer, 2015). Our results also confirm previous data on the dispensability of FgHyd5 in hyphal penetration through the water-air interface, although this protein was suggested to affect the hydrophobicity of aerial mycelia (Minenko et al., 2014). The observation that the Δ*Fghyd123* and Δ*Fghyd235* triple mutants, although to a lesser extent, still show aerial hyphae breaking through the air-liquid barrier, suggests that additional factors could be involved in this phenotype.

Forming a hydrophobic cell wall coating, HPs are reported to mediate fungal attachment to hydrophobic surfaces (Wösten et al., 1994). This feature was observed in particular for the Δ*Fghyd3* mutant as well as the Δ*Fghyd234* one, both negatively affected in the ability of germlings to attach to a hydrophobic surface.

The expression of FgHyd3 and, to a lesser extent, FgHyd2 genes is dramatically up-regulated during wheat spike infection. Thus, the contribution of the FgHyds to *T. aestivum* wheat spikes infection was investigated by point and spray inoculation methods. Only wheat infections carried out by spray inoculation, a method more similar to natural conditions in the field, showed a reduction of symptomatic spikelets after inoculation with the Δ*Fghyd2*, Δ*Fghyd3*, and Δ*Fghyd4* mutants as well as the triple mutants.

Histological analysis, performed by fluorescence microscopy, excluded a defect in the development of infection structures produced by mutant strains. Thus, our infection results could be related to the affected ability to penetrate through the water-air interface displayed by the Δ*Fghyd2* and Δ*Fghyd3* mutants, with the Δ*Fghyd3* also affected in the attachment to hydrophobic surfaces, such as those of wheat glumes. However, establishing the precise contribution of each hydrophobin to a given phenotypic trait is complicated by the observation that the non-deleted FgHyd genes are moderately to strongly up-regulated in the single Δ*Fghyd2*, Δ*Fghyd3* and especially in the triple mutants. In particular, this result could explain why we did not observe any additive effect by comparing the virulence reduction displayed by the Δ*Fghyd2* and Δ*Fghyd3* single mutants with that of the triple mutants.

Besides, the observation that the Δ*Fghyd4* mutant shows reduced virulence on wheat spikes but is not affected in the escape from the aqueous environment or in the adhesion of fungal germlings to hydrophobic surface suggests that other unknown functions of the FgHyd4 protein could affect fungal virulence during the infection process.

On the whole, our results are similar to previous data obtained with the HPs of *M. grisea*, which have been shown to be involved in the interaction with hydrophobic surfaces (Talbot et al., 1996), attachment to leaf surface (Whiteford and Spanu, 2001) and fungal pathogenicity on rice (Talbot et al., 1993; Kim et al., 2005). Differently from what observed for the HPs of *Schizophyllum commune* and *B. cinerea* (Wessels et al., 1991a; Terhem and van Kan, 2014), involved in the normal development of sexual structures, FgHyd proteins are not essential for sexual reproduction on wheat nodes, nor they are involved on asexual reproduction in axenic culture.

## Conclusion

The FgHyd2 and FgHyd3 proteins display both different and overlapping functions possibly involved in the adaptation of *F. graminearum* to culture and plant environments. FgHyd2 is involved in fungal growth, FgHyd3 in attachment to hydrophobic surfaces, both of them are involved in the hyphal ability to penetrate through the water-air interface and likely in adhesion of conidia to the wheat spike surface, thus contributing to *F. graminearum* virulence (Table 2).

**Table 2:** Phenotypes significantly affected in the FgHyd mutants.

Name	*In vitro* growth	Liquid-air interface penetration	Attachment	Wheat spray infection	Tebuconazole
Δ*Fghyd1*	n.a.	n.a.	n.a.	n.a.	n.a.
Δ*Fghyd2*	Reduced	Reduced	n.a.	Reduced	n.a.
Δ*Fghyd3*	n.a.	Reduced	Reduced	Reduced	n.a.
Δ*Fghyd4*	n.a.	n.a.	n.a.	Reduced	n.a.
Δ*Fghyd5*	n.a.	n.a.	n.a.	n.a.	n.a.
Δ*Fghyd123*	Reduced	Reduced	n.a.	Reduced	Increased sensitivity
Δ*Fghyd234*	Reduced	Reduced	Reduced	Reduced	Increased sensitivity
Δ*Fghyd235*	Reduced	Reduced	n.a.	Reduced	Increased sensitivity


*n.a., not affected.*

## Author Contributions

LS, WS, and AM-R conceived and designed the research. AQ, BH, CK, LS, and AM-R performed the experiments. AQ, BH, LS, WS, and AM-R analyzed the data. LS, FF, WS, and AM-R contributed to reagents, materials, and analysis tools. LS, FF, and WS acquired funding. AQ, LS, WS, and AM-R wrote the original draft. LS, FF, WS, and AM-R reviewed and edited draft of the manuscript.

## Conflict of Interest Statement

The authors declare that the research was conducted in the absence of any commercial or financial relationships that could be construed as a potential conflict of interest.

## Footnotes

1 ftp://ftpmips.gsf.de/fungi/Fusarium/F_graminearum_PH1_v32/

2 http://smart.embl-heidelberg.de/

3 http://myhits.isb-sib.ch/cgi-bin/motif_scan

4 http://expasy.org/tools/

5 http://primer3.ut.ee/
