# Supplementary material for: Different Hydrophobins of Fusarium graminearum Are Involved in Hyphal Growth, Attachment, Water-Air Interface Penetration and Plant Infection
Source: Front Microbiol. 2019 Apr 12;10:751. doi: 10.3389/fmicb.2019.00751 (PMC6474331; doi:10.3389/fmicb.2019.00751)
Supplement: Supplementary file 1 [file Data_Sheet_1.PDF]

SUPPLEMENTARY MATERIAL

Supplementary Figure S1. Alignment of hydrophobins amino acid sequences from Ascomycetes.

Supplementary Figure S2. Hydropathy plots of *Fusarium graminearum* hydrophobins amino acid sequences.

Supplementary Figure S3. Strategy and confirmation of FgHyd1 ORF deletion in the single and triple mutants.

Supplementary Figure S4: Strategy and confirmation of FgHyd2 ORF deletion in the single and triple mutants.

Supplementary Figure S5. Strategy and confirmation of FgHyd3 ORF deletion in the single and triple mutants.

Supplementary Figure S6. Strategy and confirmation of FgHyd4 ORF deletion in the single and triple mutants.

Supplementary Figure S7. Strategy and confirmation of FgHyd5 ORF deletion in the single and triple mutants.

Supplementary Figure S8. Conidiation assay of *Fusarium graminearum* WT, single and triple hydrophobin mutants

Supplementary Figure S9. Calcofluor white stress response of *Fusarium graminearum* hydrophobin mutants on complete and minimal medium.

Supplementary Figure S10. Fungal growth inhibition assay in the presence of glucanase or chitinase.

Supplementary Figure S11. Hydrophobins are not necessary for virulence in wheat spikes using point inoculation.

Supplementary Figure S12. *ΔFghyd2* and *ΔFghyd3* mutants of *Fusarium graminearum* are necessary for virulence independently of the wheat cultivar.

Supplementary Figure S13. Hydrophobins are not required for infection structures formation or penetration.

Supplementary Figure S14. Hydrophobin mutants are not impaired on perithecia formation or ascospores germination.

Table S1. Oligonucleotide sequences used in this study.

Table S2. Plasmids used or generated in this work.

Table S3. Comparison of gene stability of the two *Fusarium graminearum* housekeeping genes used as internal control based on their crossing point (CP) values and results provided by BestKeeper software.

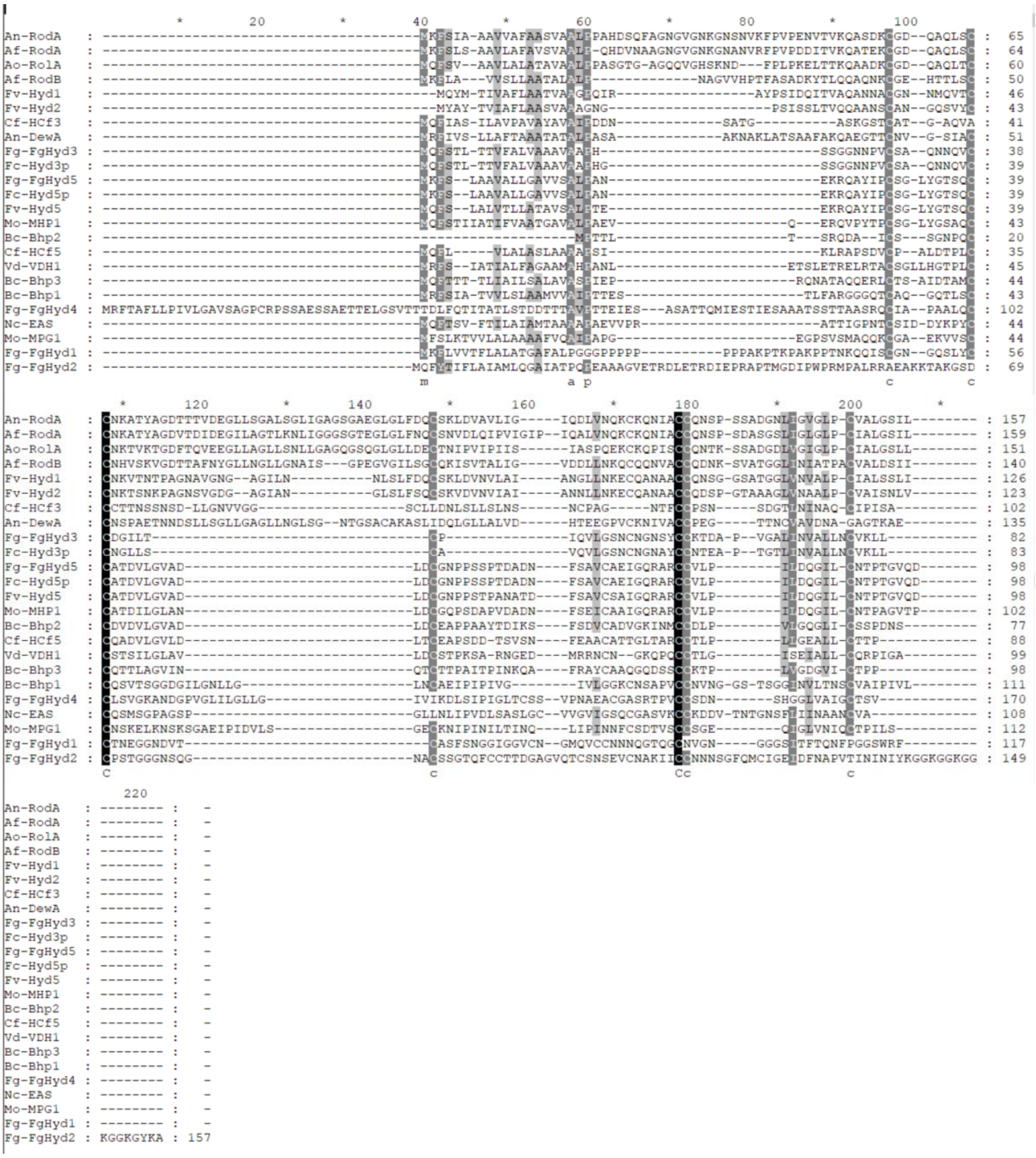

Supplementary Figure S1. Alignment of hydrophobins amino acid sequences from Ascomycetes. The alignment shows the multiple conserved cysteins on the hydrophobin fungal proteins from Ascomycetes used are: *Cladosporium fulvum* HCf3 (CAD92803); *C. fulvum* HCf5 (CAC27408); *Fusarium graminearum* FgHyd1 (FGSG_01763); *F. graminearum* FgHyd2 (FGSG_01764); *F. graminearum* FgHyd3 (FGSG_09066); *F. graminearum* FgHyd4 (FGSG_03960); *F. graminearum* FgHyd5 (FGSG_01831); *F. verticillioides* Hyd1 (Q6YF32); *F. verticillioides* Hyd2 (Q6YF31); *F. verticillioides* Hyd5 (Q6YD93); *Botrytis cinerea* Bhp1 (BC1G_15273); *B. cinerea* Bhp2 (BC1G_03994); *B. cinerea* Bhp3 (BC1G_01012); *Aspergillus oryzae* RolA (BAC65230.1); *Verticillium dahliae* VDH1 (AAY89101.1); *Magnaporthe grisea* MPG1 (P52751); *M. grisea* MHP1 (AAD18059); *F. culmorum* FcHyd3p (ABE27987.1); *F. culmorum* FcHyd5p (ABE27986.1); *Neurospora crassa* EAS (EAA34064.1); *Claviceps purpurea* CPPH1 (CAD10781.1); *Aspergillus nidulans* RodA (AAA33321.1); *A. nidulans* DewA (AAC13762.1); *A. fumigatus* RodA (AAB60712.1) and *A. fumigatus* RodB (EAL91055.1). The corresponding accession numbers were obtained from the NCBI database (http://www.ncbi.nlm.nih.gov/).

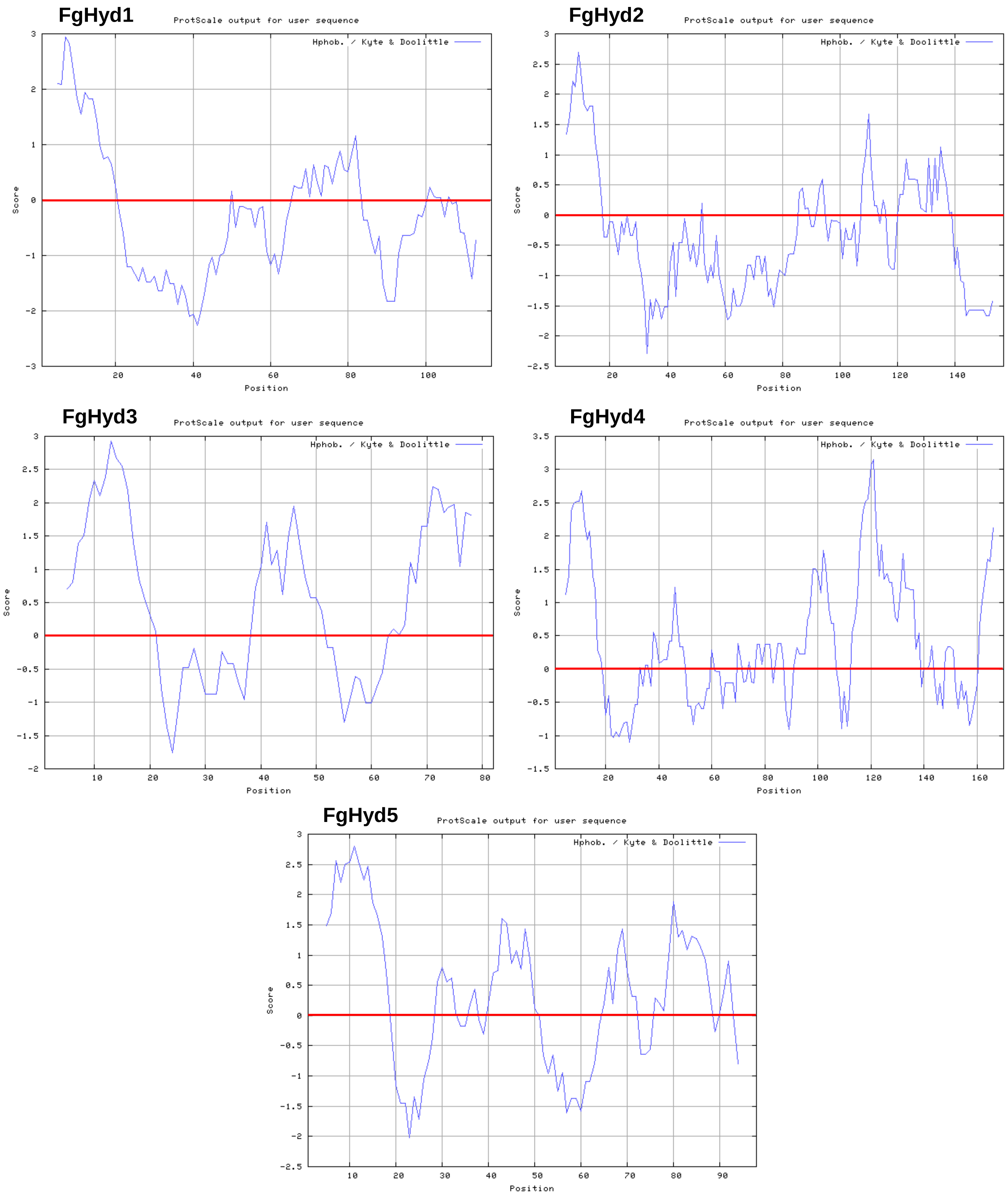

Supplementary Figure S2. Hydropathy plots of *Fusarium graminearum* hydrophobins amino acid sequences. The hydropathy score was calculated with ProtScale software (http://expasy.org/tools/) by using the Kyle & Doolittle aa scale. Hydrophobic aa show positive peaks with values above 0 whereas hydrophilic aa show negative peaks. While FgHyd1 and FgHyd2 show less hydrophobic residues, FgHyd3, FgHyd4 and FgHyd5 present higher hydropathy score.

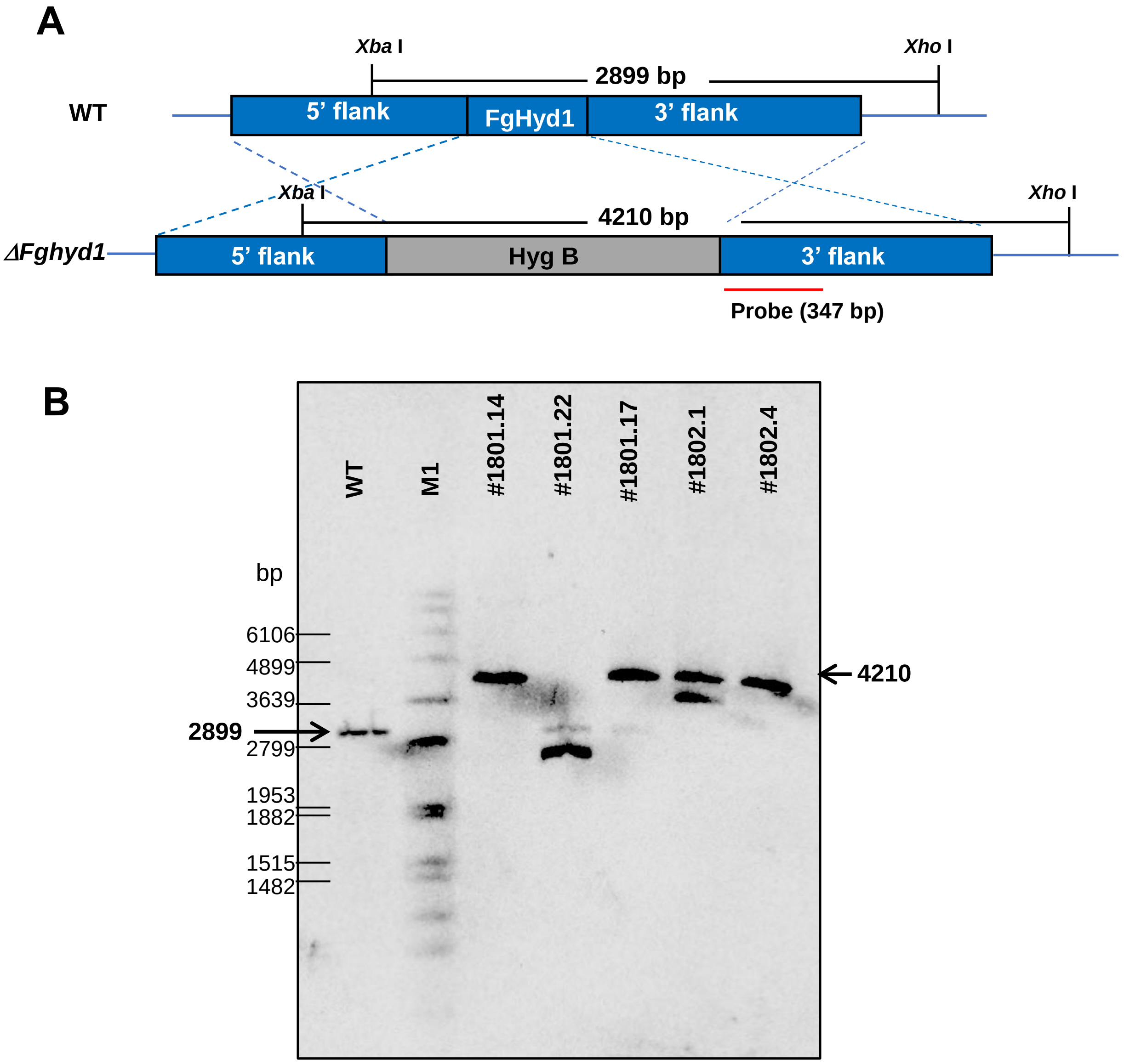

Supplementary Figure S3. Strategy and confirmation of FgHyd1 ORF deletion in the single and triple mutants. (**A**) Schematic representation of FgHyd1 gene deletion and Southern blot strategies. FgHyd1 ORF was replaced with the hygromycin B (Hyg B) resistance cassette. *Xho*I and *Xba*I enzymes were used for genomic DNA restriction of the WT strain, single and triple mutants. Red bar = probe used (**B**) Southern blot showing bands at 4210 bp confirming double homologous recombination and single integration of the deletion construct in the single *ΔFghyd1* (#1801.14 and 17) and triple *ΔFghyd123* (#1802.4) deletion mutants or 2899 bp for WT and ectopic integration (#1801.22) on the single mutant. M1: DNA molecular weight Dig-marker VII. Triple mutant #1802.1 shows two integrated copies.

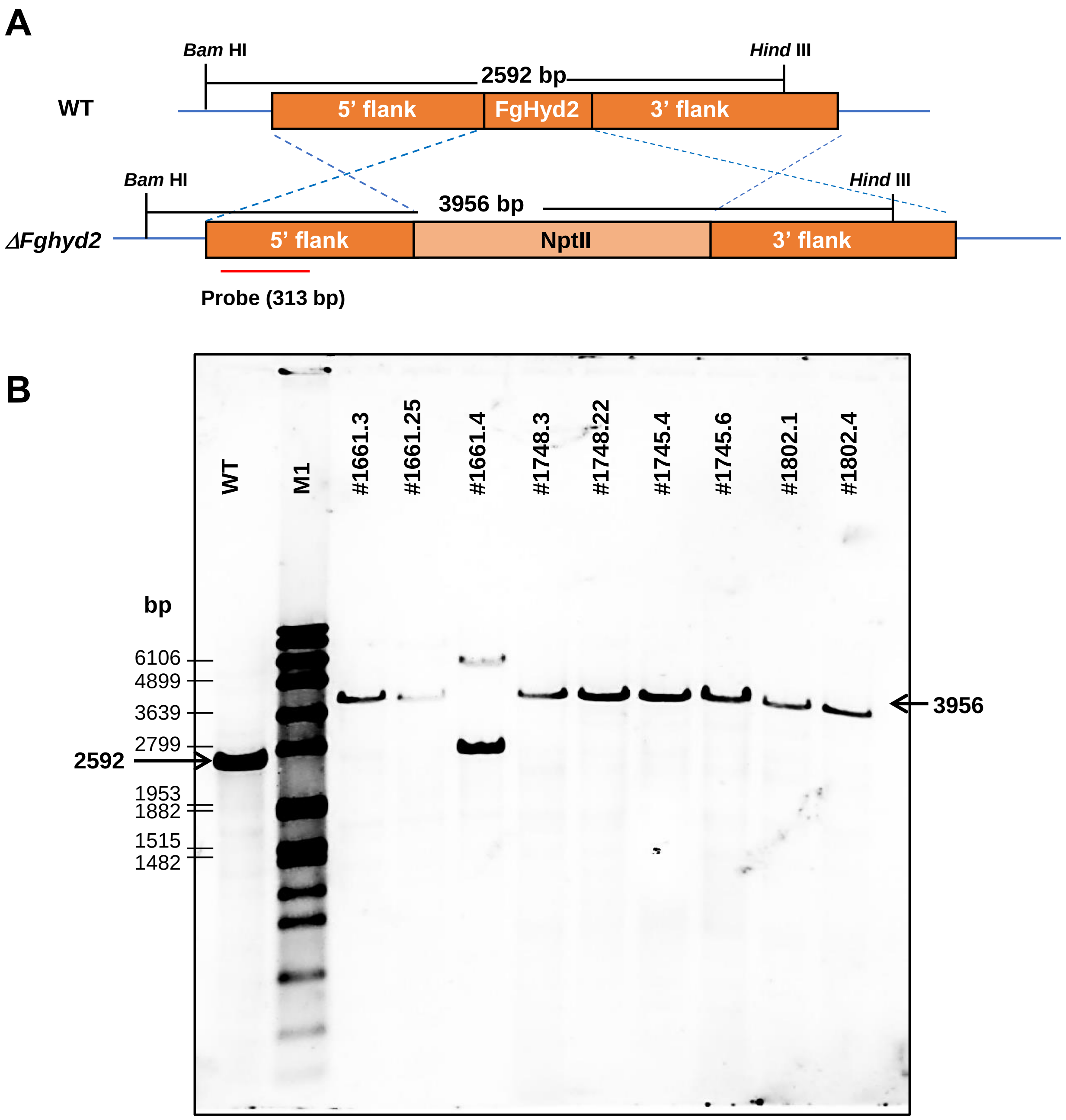

Supplementary Figure S4: Strategy and confirmation of FgHyd2 ORF deletion in the single and triple mutants. (**A**) Schematic representation of FgHyd2 gene deletion and Southern blot strategies. FgHyd2 ORF was replaced with the geneticin (NptII: neomycin phosphotransferase) resistance cassette. *Bam*HI and *Hind*III enzymes were used for genomic DNA restriction of the WT strain, single and triple mutants. Red bar = probe used (**B**) Southern blot showing bands at 3956 bp confirming double homologous recombination and single integration of the deletion construct in the single *ΔFghyd2* mutants (#1661.3 and 25), triple mutants *ΔFghyd235* (#1748.3 and 22), triple mutants *ΔFghyd234* (#1745.4 and 6) and triple mutants *ΔFghyd123* (#1802.1 and 4). Bands at 2592 bp for WT and ectopic integration in the single mutant #1661.4 which shows the integrated copy at 6100 bp. M1: DNA molecular weight Dig-marker VII.

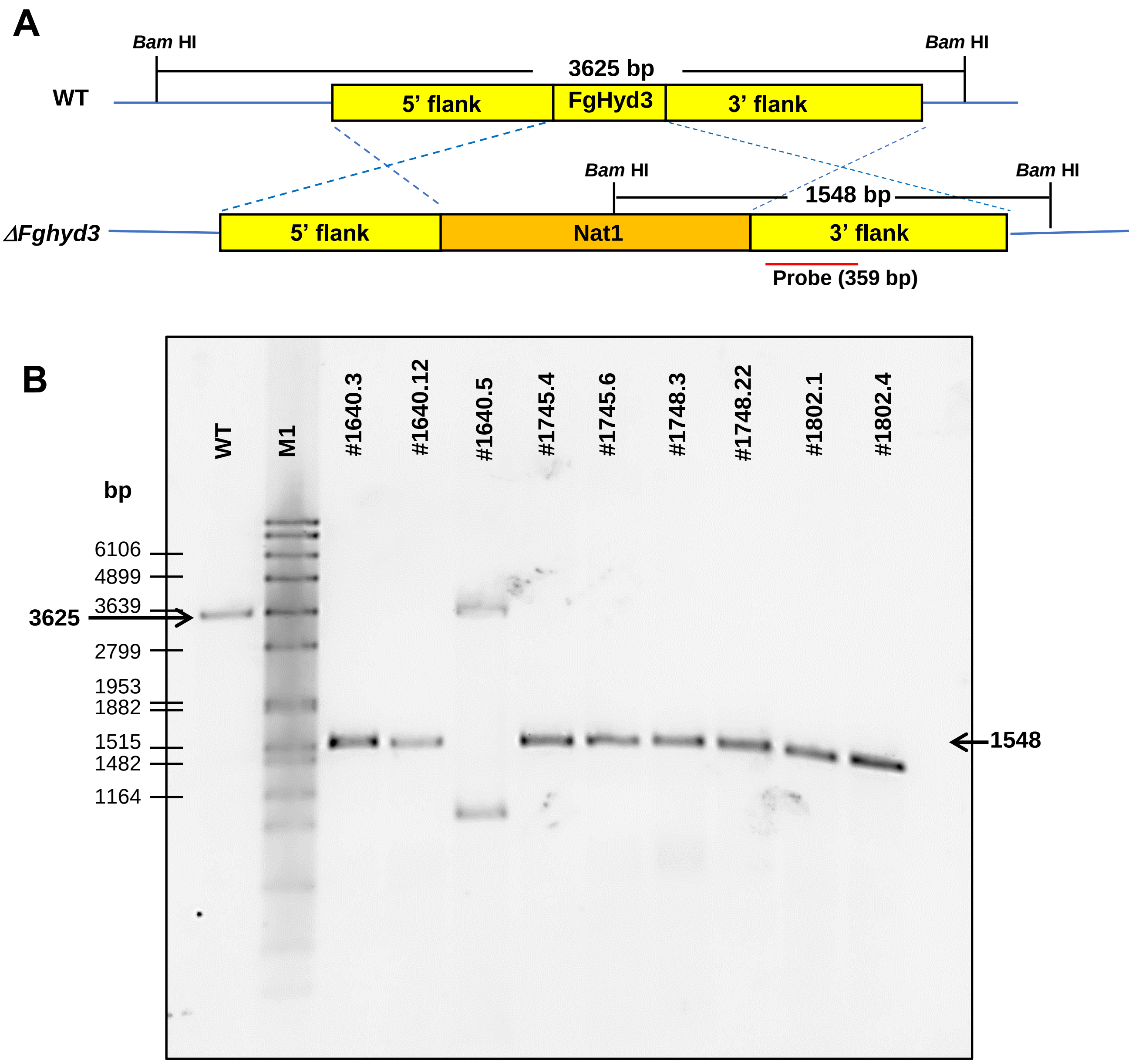

Supplementary Figure S5. Strategy and confirmation of FgHyd3 ORF deletion in the single and triple mutants. (**A**) Schematic representation of FgHyd3 gene deletion and Southern blot strategies. FgHyd3 ORF was replaced with the nourseothricin (Nat1) resistance cassette. *Bam*HI enzyme was used for genomic DNA restriction of the WT strain, single and triple mutants. Red bar = probe used (**B**) Southern blot showing bands at 1548 bp confirming double homologous recombination and single integration of the deletion construct in the single *ΔFghyd3* mutants (#1640.3 and 12), triple mutants *ΔFghyd234* (#1745.4 and 6), triple mutants *ΔFghyd235* (#1748.3 and 22) and triple mutants *ΔFghyd123* (#1802.1 and 4). Bands at 3625 bp for WT and ectopic integration in the single mutant #1640.5. M1: DNA molecular weight Dig-marker VII.

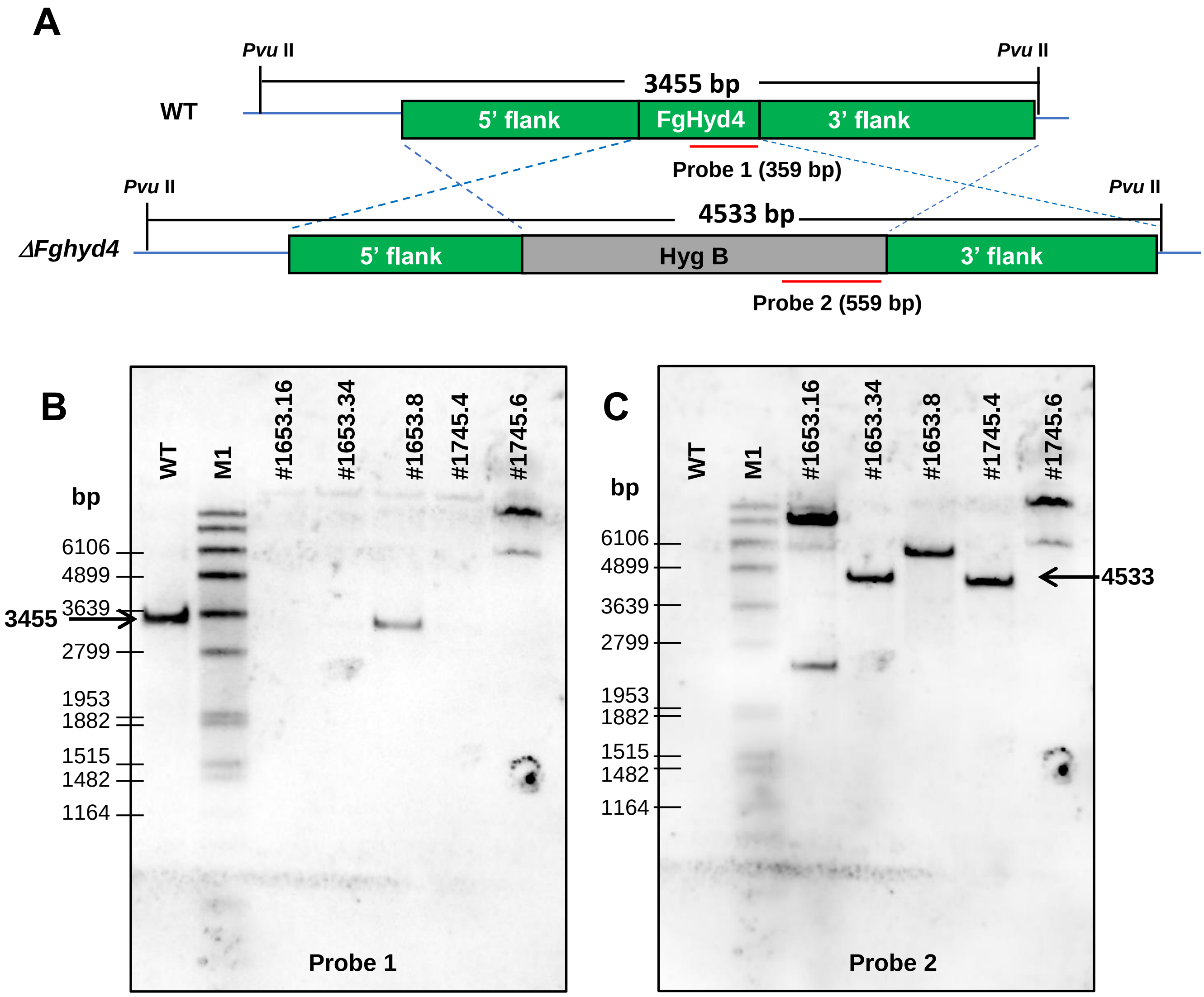

Supplementary Figure S6. Strategy and confirmation of FgHyd4 ORF deletion in the single and triple mutants. (**A**) Schematic representation of FgHyd4 gene deletion and Southern blot strategies. FgHyd4 ORF was replaced with the hygromycin B (Hyg B) resistance cassette. *Pvu*II enzyme was used for genomic DNA restriction of the WT strain, single and triple mutants. Red bars= probes used (**B**) Southern blot using probe 1 (inside of the FgHyd4 ORF) showing the lack of bands confirming double homologous recombination of the deletion construct in the single *ΔFghyd4* mutants (#1653.16 and 34), triple mutants *ΔFghyd234* (#1745.4 and 6). Bands at 3455 bp for WT and ectopic integration in the single mutant #1653.8. (**C**) Southern blot using probe 2 (inside of the Hyg B resistance cassette) showing bands at 4533 bp confirming double homologous recombination and single integration of the deletion construct in the single *ΔFghyd4* mutant (#1653.34) and triple mutant *ΔFghyd234* (#1745.4). Lack of bands for WT, ectopic integration in the single mutant #1653.8 and multiple integrations in the single mutant #1653.16 and triple mutant #1745.6. M1: DNA molecular weight Dig-marker VII.

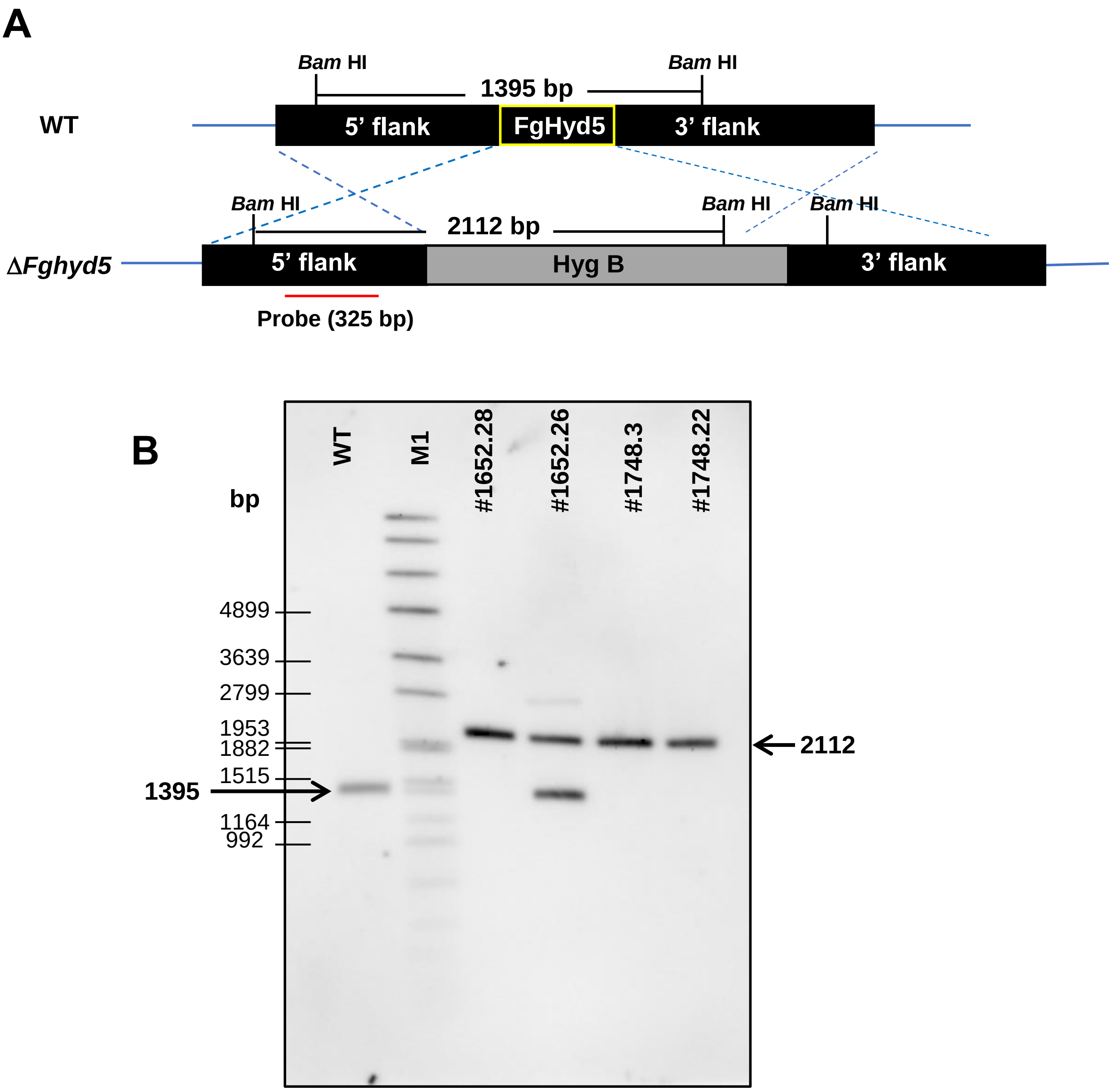

Supplementary Figure S7. Strategy and confirmation of FgHyd5 ORF deletion in the single and triple mutants. (**A**) Schematic representation of FgHyd5 gene deletion and Southern blot strategies. FgHyd5 ORF was replaced with the hygromycin B (Hyg B) resistance cassette. *Bam*HI enzyme was used for genomic DNA restriction of the WT strain, single and triple mutants. Red bar = probe used (**B**) Southern blot showing bands at 2112 bp confirming double homologous recombination and single integration of the deletion construct in the single *ΔFghyd5* mutant (#1652.28) and triple mutants *ΔFghyd235* (#1748.3 and 22). Bands at 1395 bp for WT and ectopic integration in the single mutant #1652.26. M1: DNA molecular weight Dig-marker VII.

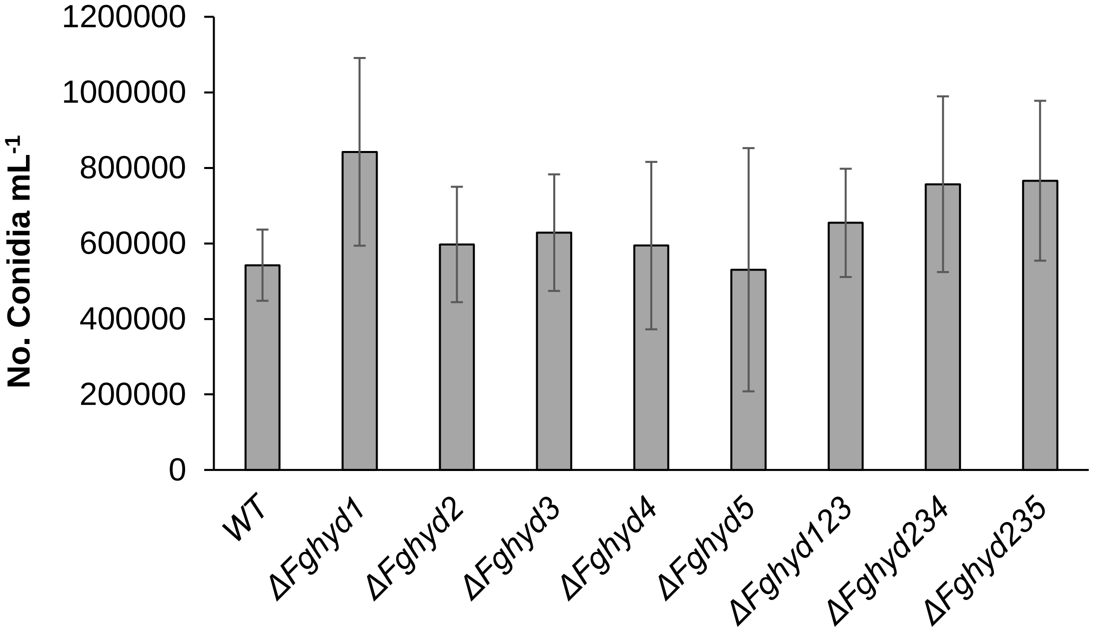

Supplementary Figure S8. Conidiation assay of *Fusarium graminearum* WT, single and triple hydrophobin mutants. Agar plugs of *F. graminearum* WT, single and triple mutants were used to inoculate 30 mL of carboxymethyl cellulose (CMC; Sigma-Aldrich) liquid medium. Conidia were counted after 6 days of cultures as reported in Sella et al. (2016). Data represent the mean ± standard error (indicated by bars) of at least three independent experiments. Statistical analysis was calculated with respect to the WT using one way-Anova Bonferroni-Holm. The experiment was performed by using two independent knock-out mutants for each gene obtaining similar results.

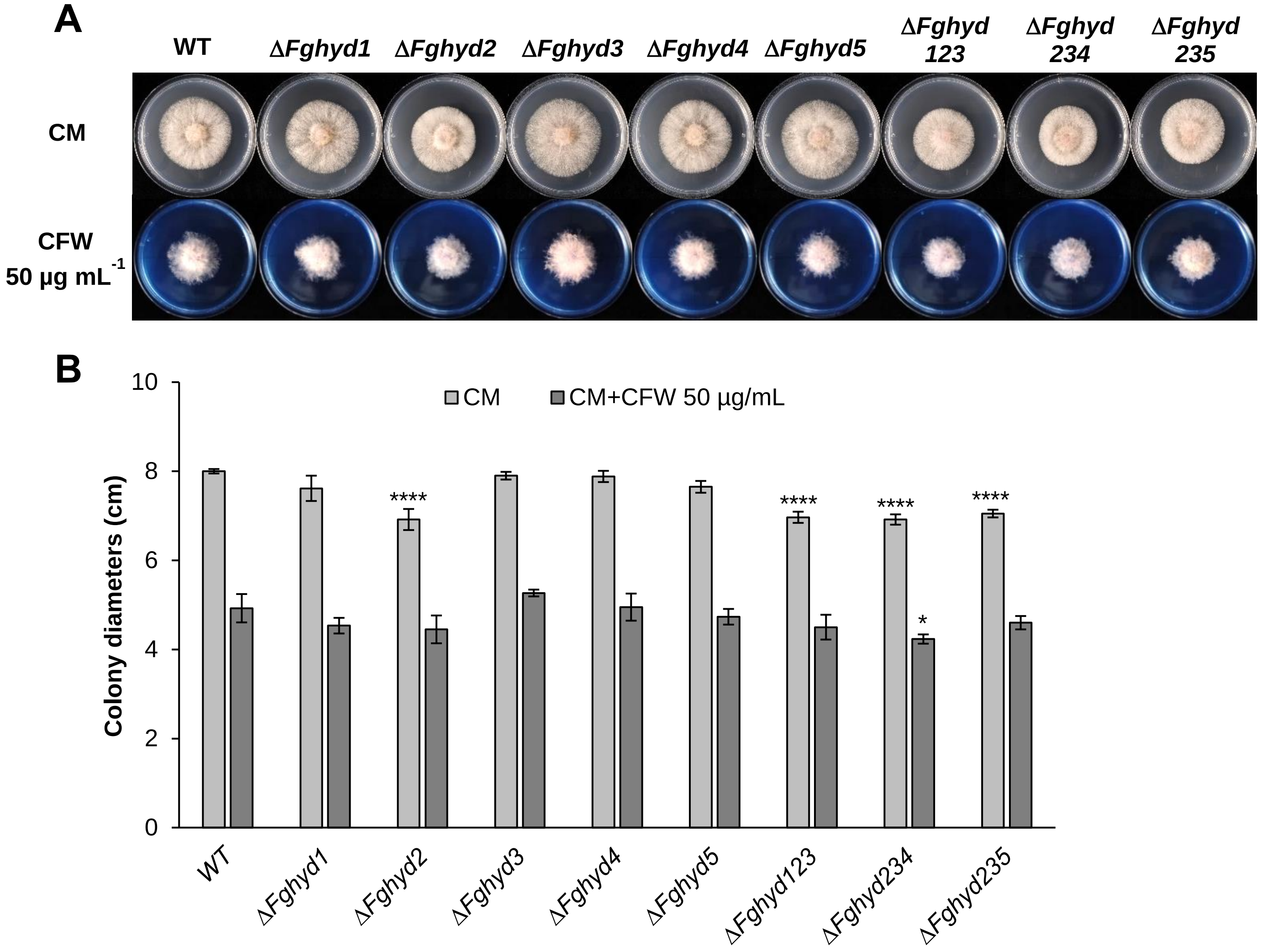

Supplementary Figure S9. Calcofluor white stress response of *Fusarium graminearum* hydrophobin mutants on complete and minimal medium. Plates containing CM or CM supplemented with 50 µg mL^{-1} of calcofluor white (CFW) were inoculated with 4-mm plugs of actively growing mycelia of the WT strain, single or triple deletion mutants. Plates were incubated for two days at 28 °C in the dark. (**A**) Pictures representative of each treatment were taken at 2 dpi. (**B**) The susceptibility to stress was estimated by measuring mycelia diameters after 2 days. Error bars indicate standard deviation calculated from data representative of 2 biological experiments and 3 experimental replicates. Statistical analysis of each treatment was calculated with respect to the WT using one way-Anova Bonferroni-Holm (significance: * $p<0.05$, **** $p<0.0001$).

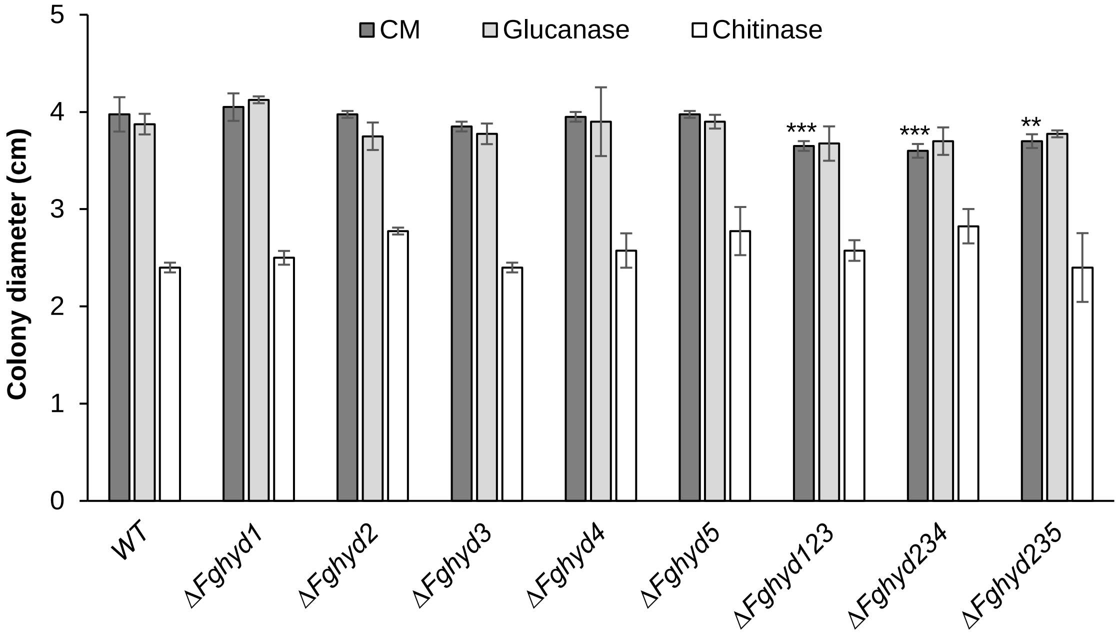

Supplementary Figure S10. Fungal growth inhibition assay in the presence of glucanase or chitinase. Agar disks (7-mm diameter) of actively growing mycelium of *F. graminearum* WT, single and triple mutant strains were inoculated on 10-mm diameter sterile paper disks imbibed with CM or 4 U of β-1,3-glucanase enzymatic solution from *Trichoderma longibrachiatum* or 1 U of *Trichoderma viride* chitinase enzymatic solution and placed on CM agar plates. Growth was measured after 48 hours at 28 °C in the dark. Error bars indicate standard deviation calculated from data representative of 2 biological experiments. Statistical analysis of each treatment was calculated with respect to the WT using one way-Anova Bonferroni-Holm (significance: ** $p<0.01$, *** $p<0.001$).

A

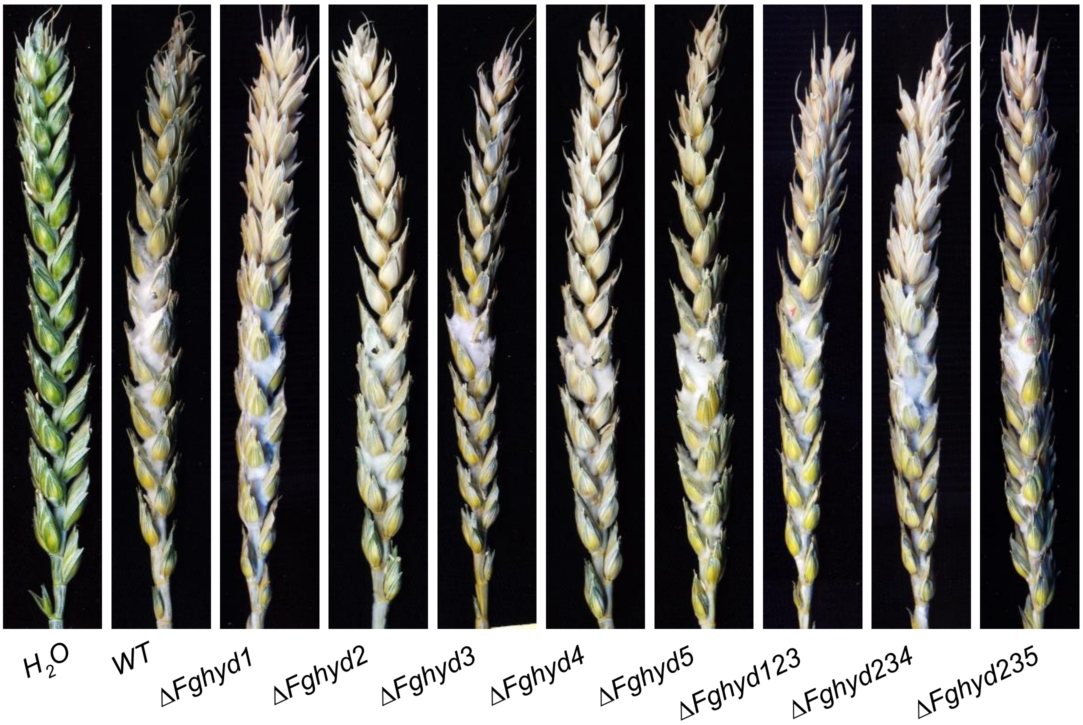

B

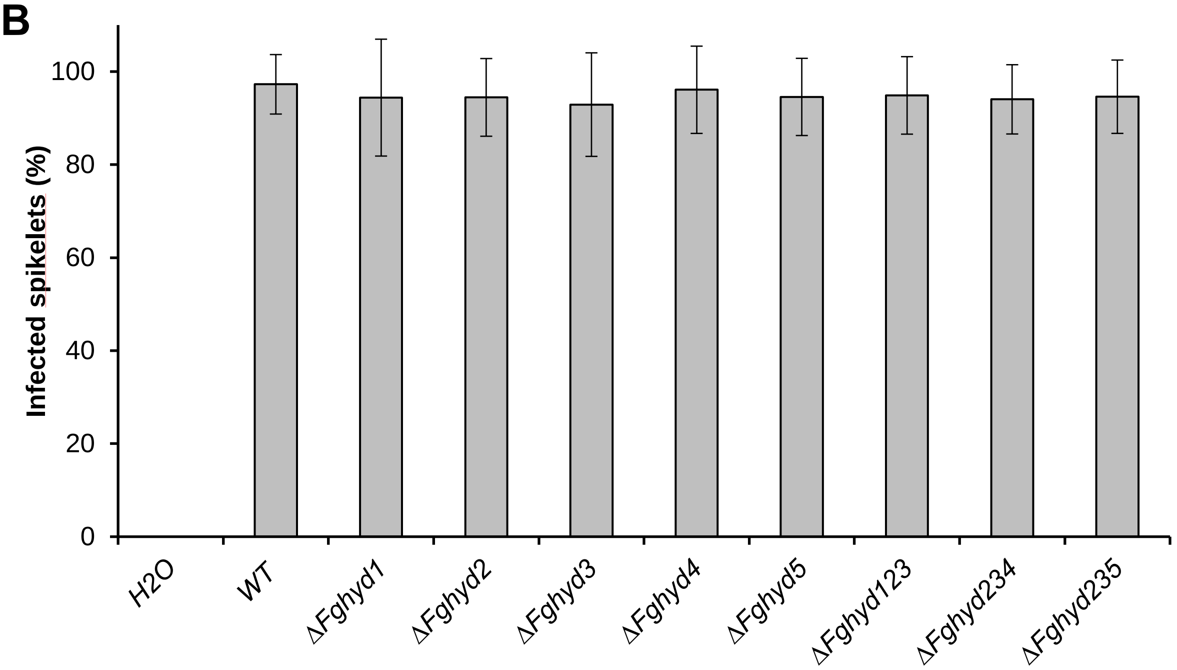

Supplementary Figure S11. Hydrophobins are not necessary for virulence in wheat spikes using point inoculation. Wheat spikes (cv. Nandu) were inoculated with 10 µL of a conidial suspension containing 2×10^4 conidia mL^{-1} of the WT or hydrophobins deletion mutants. Pictures and percentage of infection were produced at 21 dpi. (**A**) All hydrophobin deletion mutants produced full infection on the spikes similar to the WT. Water was used as a negative control. (**B**) Percentage of infection was determined by counting the amount of infected spikelets where full infected was 100%**.** Error bars indicate standard deviations calculated from 6 spikes for each treatment and 3 independent experiments (n = 18). The experiment was performed by using two independent knock-out mutants for each gene obtaining similar results.

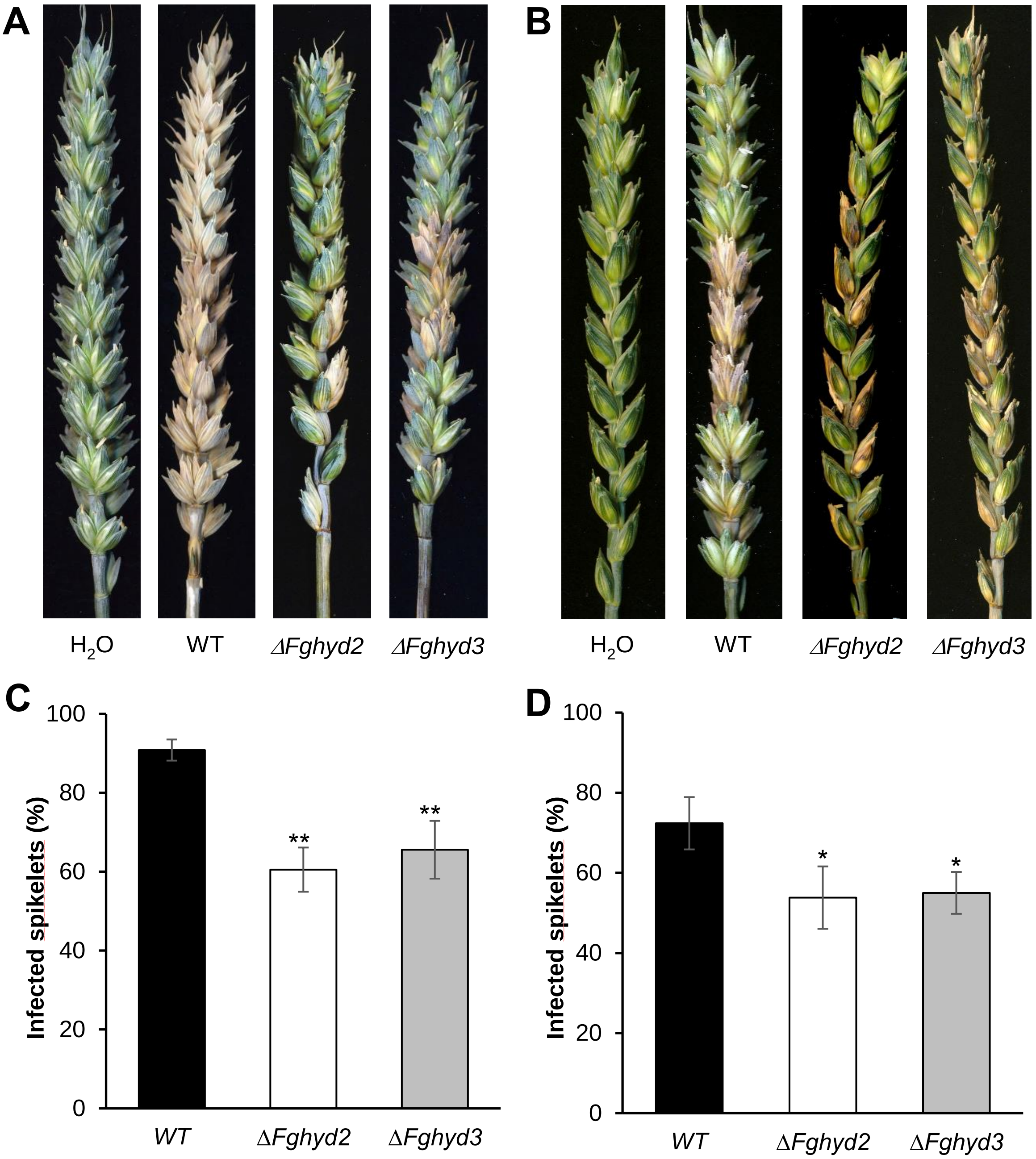

Supplementary Figure S12. *ΔFghyd2* and *ΔFghyd3* mutants of *Fusarium graminearum* are necessary for virulence independently of the wheat cultivar. Wheat spikes of the susceptible cultivar Nandu (**A**) or the semi-resistant cultivar Amaretto (**B**) were sprayed twice with 100 µL of a conidial suspension containing 500 conidia μL^{-1} of the WT strain, Δ*Fghyd2* or Δ*Fghyd3* single mutants. After 24 h the spikes were sprayed twice with 2 mL of water. The spikes sprayed with the single mutants developed less symptoms than the WT in either wheat cultivars Nandu or Amaretto. Water was used as mock inoculation. Disease symptoms were assessed at 21 dpi by counting the number of visually diseased spikelets on wheat cultivar Nandu (**C**) or Amaretto (**D**). Infected spikelets are expressed as percentage of symptomatic spikelets on total number of spikelets of the respective head. Data represent the mean ± standard error (indicated by bars) of ten independent infection experiments. Statistical analysis was calculated with respect to the WT using one way-Anova Bonferroni-Holm (significance: * $p<0.05$, ** $p<0.01$).

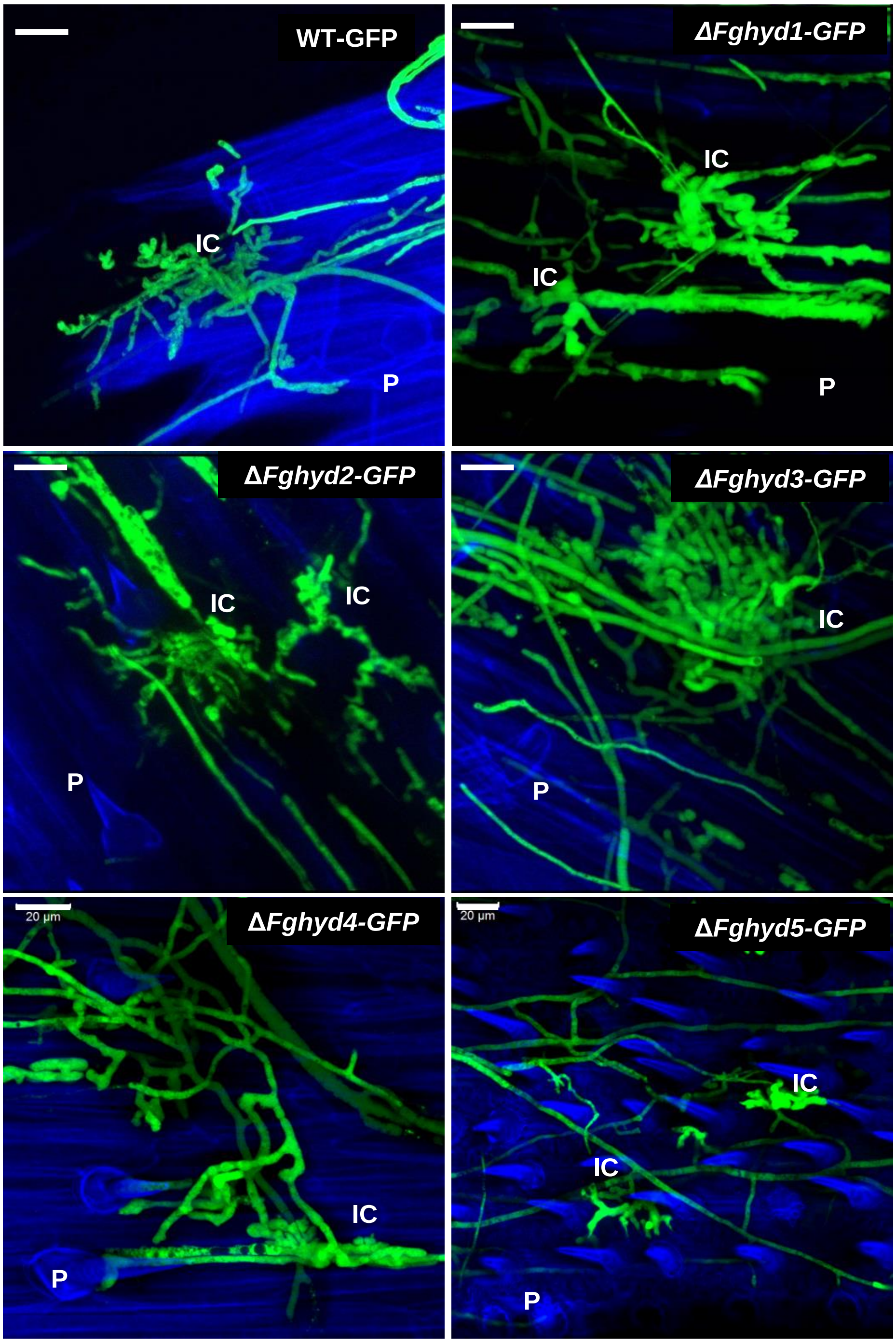

Supplementary Figure S13. Hydrophobins are not required for infection structures formation or penetration. Infection structures were produced by adding a drop of 5 µL with a conidial suspension of 2×10^{4} conidia mL^{-1} of the WT or the single deletion mutants expressing GFP constitutively on wheat paleae (cv. Nandu). All hydrophobin single deletion mutants produced infection structures similar to the WT. Two independent experiments with 8 paleae per treatment and experiment were produced (n = 16). Detection of infection structures was performed at 5 dpi using the Zeiss LSM 780 laser scanning microscope (LSM). Images are maximum intensity projection of 20 pictures each. IC: infection cushion, P: plant cell. Scale bar = 20 µm.

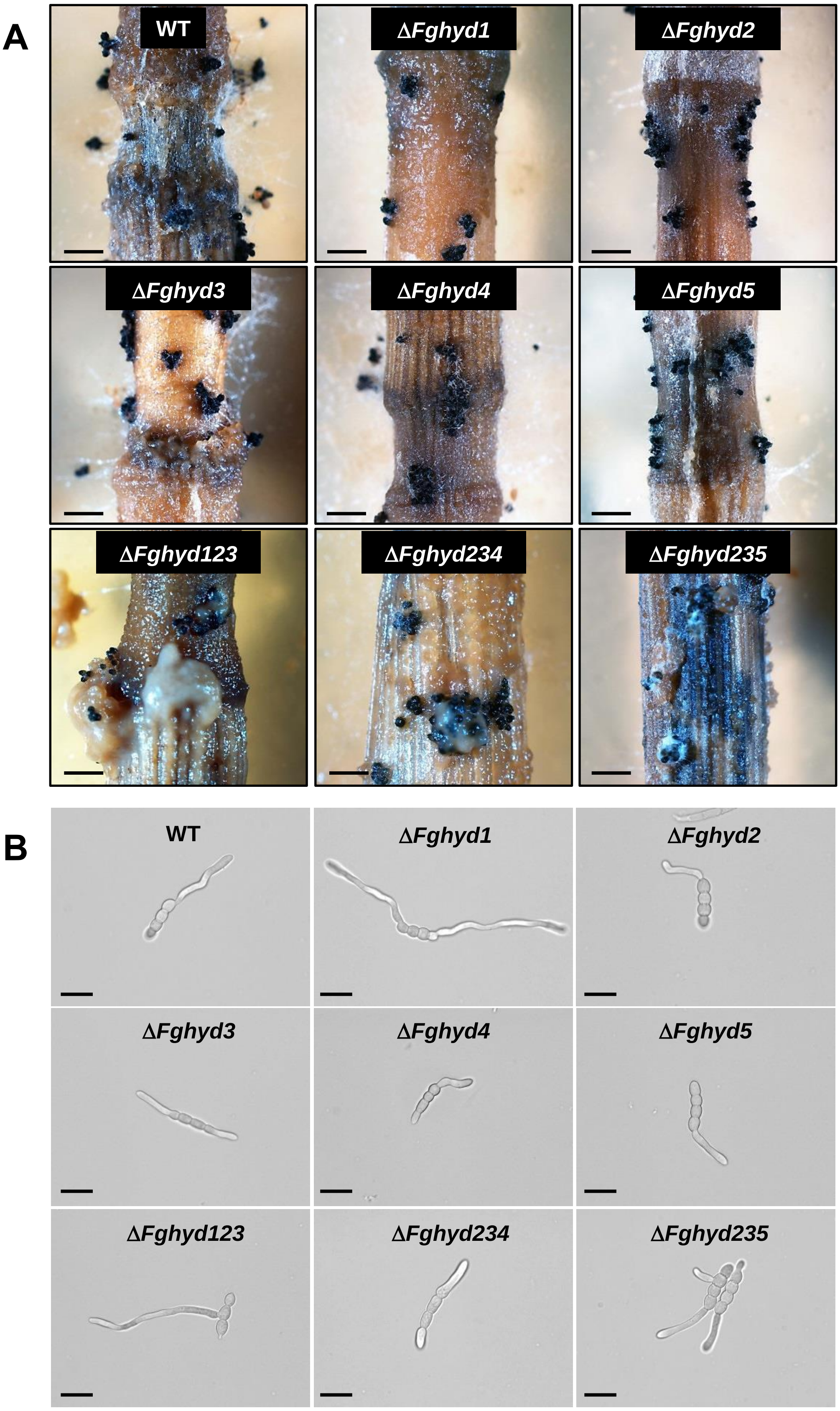

Supplementary Figure S14. Hydrophobin mutants are not impaired on perithecia formation or ascospores germination. All hydrophobin deletion mutants formed perithecia on wheat nodes similarly to the WT. Additionally, ascospores from all strains

germinated similarly to the WT. (**A**) Wheat nodes inoculated with the WT or hydrophobin deletion mutants were placed on a water-agar plate and incubated on a chamber with UV and white light during 35 days. Pictures of perithecia were taken after 35 days using a stereo microscope (Leica- ZDFIII). 6 plates containing 5 wheat nodes were used per strain. Scale bar= 2 mm. The experiment was performed by using two independent knock-out mutants for each gene obtaining similar results. (**B**) Ascospores taken from perithecia were germinated on CM for 4 h at 28 °C in the dark. Bright field pictures were taken with a Z1 fluorescence microscope (Nikon). Scale bar = 20µm.

Table S1. Oligonucleotide sequences used in this study.

Primer	Sequence (5´-> 3´)	Application
1F-Fghyd1-Vect	tgtaaaacgacggccagtgagcgcgcgtACATGCACAGCAGTTGAACAAC	FgHyd1 deletion construct (FGSG_ 01763)
2R-Fghyd1-Hyg	aaaggaatagagtagatgccgaccgaacGAGGGTTGGAAAAGTGTTTGTC	
3F-Fghyd1-Hyg	agtcaatgctacatcacccacctcgctcCACATCAACCTTTTTACTGGGA	
4R-Fghyd1-vect	ctctagaactagtggatcccccgggctgGAATGGAGTAGGGAATGAATGG	
5F-Fghyd1-out	GTCAAGTCGATTAAGGGCTAG	Primers outside of the construct for knock-out checking
6R-Fghyd1-out	GGTGCGATTGGCGTTTTTATG	
7F-Fdhyd1-int	CTCTACTGCTGCACCAACGAA	Internal primers for knock-out checking or qPCR
8R-Fghyd1-int	CTGGGTGAAGGTGATGGAGC	
9F-Fghyd1-pro	CATGGATCGACACTTACTCAC	Probe
10R-Fghyd1-pro	GGTAGAACAGATAGCTCAAGG	
1F-Fghyd2-Vect	tgtaaaacgacggccagtgagcgcgcgtTGCATGTAACAGTCAGGCCAC	FgHyd2 deletion construct (FGSG_ 01764)
2R-Fghyd2-Gen	ctatcgccttcttgacgagttcttctgaGATGATGATGGTTGATAGTAGC	
5F-Fghyd2-Gen	caggtaggccgaataacttgcacaaattggGTCGAGACACCGCGTTGCTT	
6R-Fghyd2-vect	cacacaggaaacagctatgaccatgattaTGATGCTCTAGCCGTCTTTGG	
7F-Fdhyd2-out	GCATGGCCTGTCTGTCTCAA	Primers outside of the construct for knock-out checking
8R-Fghyd2-out	TCAGAGAAGTGGCTATTGGG	
9F- Fdhyd2-int	CAAGAAGACTGCCAAAGGTAG	Internal primers for knock-out checking or qPCR
10R- Fdhyd2-int	CCACCCTTGTAGATGTTGATG	
11F-Fghyd2-pro	GCCGAAGTACAAAGACCAACC	Left flanking probe
12R-Fghyd2-pro	GACCAACATGCATGCTACCG	
1F-Fghyd3-Vect	ggcccccccctcgaggtcgacggtatcgatCAGACGTCGAATGAGAGCCGC	FgHyd3 deletion construct

2R-Fghyd3-Nat<br>3F-Fghyd3-Nat<br>4R-Fghyd3-vect	acatgagcatgccctgcccctgagcggccGTGTTGAAAGTGTTGGAGGAT<br>cccgaatcgggaatgcggctctagagtagTGTTGTAAGTTTCACTCACAC<br>gctctagaactagtggatcccccgggctgACCAGAAATGTTGGTCGCTTG	(FGSG_09066)
5F-Fdhyd3-out<br>6R-Fghyd3-out	CGATCCCTTCTACAAATAAACGTC<br>GATAAAGGTTCCTCAAGGCCC	Primers outside of the construct for knock-out checking
7F- Fdhyd3-int<br>8R- Fdhyd3-int	ATGCAGTTCTCTACTCTCACC<br>GTGTGAGTGAAACTTACAACA	Internal primers for knock-out checking or qPCR
9F-Fghyd3-pro<br>10R-Fghyd3-pro	CACTCACACACTCTACAAACG<br>GGAAATGTGTGCAGATGTCAG	Right flanking probe
1F-Fghyd4-Vect<br>2R-Fghyd4-Hyg<br>3F-Fghyd4-Hyg<br>4R-Fghyd4-vect	tgtaaaacgacggccagtgagcgcgcgtGAATATGAAGGTTTTGCTGGTG<br>caaaggaatagagtagatgccgaccgaacTTGGTGGATCAGGTGGCTTTC<br>agtcaatgctacatcacccacctcgctcAGATAAAGCCACTACTGAACTG<br>ctctagaactagtggatcccccgggctgTAGCAGTTTGTCTTATTGGTGG	FgHyd4 deletion construct (FGSG_03960)
5F-Fdhyd4-out<br>6R-Fghyd4-out	TGGACGATGGGAAGATATGGG<br>GGGGATAACATGATTGACGTGA	Primers outside of the construct for knock-out checking
7F- Fdhyd4-int<br>8R- Fdhyd4-int	CTTCCACGACTGCTGCTTCT<br>GTACAGCCAATAGCAACAAGG	Internal primers for knock-out checking or qPCR
9F-Fghyd4-pro<br>10R-Fghyd4-pro	TACTGAACTGGTTGGCTGCAT<br>CTACAATCTTCCCTCACTTCC	Probe
1F-Fghyd5-Vect<br>2R-Fghyd5-Hyg<br>3F-Fghyd5-Hyg<br>4R-Fghyd5-vect	tgtaaaacgacggccagtgagcgcgcgtGGTCAACGTTTACGATCCCAG<br>aaaggaatagagtagatgccgaccgaacAGAGTGATTGGTTTAGGAGAGT<br>agtcaatgctacatcacccacctcgctcTCCTCTGCAATACCCCTACTG<br>ctctagaactagtggatcccccgggctgGTATCATAGCGTACAACTGTGT	FgHyd5 deletion construct (FGSG_01831)
5F-Fdhyd5-out<br>6R-Fghyd5-out	GTGTTAGGGGAGATTCAAGGA<br>TGGGGACGACAAGGATGCTG	Primers outside of the construct for knock-out checking
7F- Fdhyd5-int<br>8R- Fdhyd5-int	AACGAGAAGCGACAGGCCTA<br>GATGGGGAGGACACAGCAG	Internal primers for knock-out checking or qPCR
9F-Fghyd5-pro<br>10R-Fghyd5-pro	CCGCACTTTAAGACAGCCACT<br>CGAAATCGCCACAGCAAGTTA	Probe

1F-Hyg<br>2R-Hyg	GAGCGAGGTGGGTGATGTAG<br>CGGTCGGCATCTACTCTATTC	Hygromicin (Hyg) resistance cassette
1F-NptII<br>3R-NptII	GCCAGTTGTTCCCAGTGATCT<br>GCGAGGTCCAATGCATTAATG	Geneticin (NptII) resistance cassette
1F- Nat<br>5R-Nat	CTAAAGGGAACAAAAGCTGGAG<br>ACGATATCGAATTCCTGCAGG	Nourseothricin (Nat) resistance cassette
qF-FgTUB<br>qR-FgTUB	TGTCGACGACCAGTTCTCAGC<br>CGATGTCGGCGTCTTGGTAT	qPCR expression analyses
q7F-FgeIF5A<br>q8R-FgeIF5A	CTGGCAAGAAGTACGAGGAT<br>GCCCATAGAGGTGAGGACAAT	
F: forward primer; R: reverse primer. Lowercase: overhangs for fusion.		

Table S2. Plasmids used or generated in this work.

Name	Restricted with	Use	References
pRS426	*Eco*RI and *Hin*dIII	Yeast cloning method	Christianson et al., 1992
pNRI	Not restricted	Nourseothricine (Nat) selectable marker	Malonek et al. 2004
pGEM-Hyg	Not restricted	Hygromicine (Hyg) selectable marker	Maier et al., 2005
pII99	Not restricted	Geneticin (NptII) selectable marker	Beck et al., 1982
pIGPAPA	*Hin*dIII	GFP constitutive with Hyg selectable marker (PgpdA:GFP:hyg)	Lee et al., 2003
pBH-GFP	*Hin*dIII	GFP constitutive with NptII selectable marker (PgpdA:GFP:NptII)	This study
pALM-Fghyd1::Hyg	*PCR product*	Fghyd1 deletion construct	This study
pALM-Fghyd2::NptII	*Hin*dIII, *Age*I	Fghyd2 deletion construct	This study
pJB-Fghyd3::Nat	*Xho*I, *Sac*II	Fghyd3 deletion construct	This study
pALM-Fghyd4::Hyg	*Eco*9I, *Not*I	Fghyd4 deletion construct	This study
pALM-Fghyd5::Hyg	*Xho*I, *Xba*I	Fghyd5 deletion construct	This study

Table S3. **Comparison of gene stability of the two *Fusarium graminearum* housekeeping genes used as internal control based on their crossing point (CP) values and results provided by BestKeeper software.**

CP data of housekeeping Genes		
	**Tub (**FGSG_06611)	**eIF5A** (FGSG_01955)
n	40	40
geo Mean [CP]	22.96	22.38
ar Mean [CP]	22.98	22.40
min [CP]	21.53	21.63
max [CP]	24.85	24.30
**std dev [± CP]**	**0.71**	**0.62**
CV [% CP]	3.11	2.77
min [x-fold]	-2.70	-1.69
max [x-fold]	3.71	3.77
std dev [± x-fold]	1.64	1.54
[a]**BestKeeper vs.**	**HKG 1**	**HKG 2**
coeff. of corr. [r]	0.796	0.711
p-value	0.001	0.001

[a]Repeated Pair-wise Correlation Analysis of BestKeeper vs. Housekeeping Genes
